# Genetic-guided pharmacotherapy for venous thromboembolism: a systematic and critical review of economic evaluations

**DOI:** 10.1038/s41397-021-00243-7

**Published:** 2021-06-15

**Authors:** Ka Keat Lim, Rositsa Koleva-Kolarova, Philip Chowienczyk, Charles D. A. Wolfe, Julia Fox-Rushby

**Affiliations:** 1 School of Population Health and Environmental Sciences, Faculty of Life Sciences and Medicine, King’s College London, London, UK; 2 National Institute for Health Research (NIHR) Biomedical Research Centre, Guy’s and St Thomas’ NHS Foundation Trust and King’s College London, London, UK; 3 Health Economics Research Centre, Nuffield Department of Population Health, University of Oxford, Oxford, UK; 4 Cardiovascular Division, Department of Clinical Pharmacology, King’s College London School of Medicine, St Thomas’ Hospital, London, UK; 5 National Institute for Health Research (NIHR) Collaboration for Leadership in Applied Health Research and Care (ARC) South London, London, UK

**Keywords:** Health services, Outcomes research

## Abstract

Despite the known contributions of genes, genetic-guided pharmacotherapy has not been routinely implemented for venous thromboembolism (VTE). To examine evidence on cost-effectiveness of genetic-guided pharmacotherapy for VTE, we searched six databases, websites of four HTA agencies and citations, with independent double-reviewers in screening, data extraction, and quality rating. The ten eligible studies, all model-based, examined heterogeneous interventions and comparators. Findings varied widely; testing was cost-saving in two base-cases, cost-effective in four, not cost-effective in three, dominated in one. Of 22 model variables that changed decisions about cost-effectiveness, effectiveness/relative effectiveness of the intervention was the most frequent, albeit of poor quality. Studies consistently lacked details on the provision of interventions and comparators as well as on model development and validation. Besides improving the reporting of interventions, comparators, and methodological details, future economic evaluations should examine strategies recommended in guidelines and testing key model variables for decision uncertainty, to advise clinical implementations.

## Introduction

Venous thromboembolism (VTE), comprising deep vein thrombosis (DVT) and pulmonary embolism (PE), is a major cardiovascular disease (CVD) [1]. Patients with previous VTE are at high risk of recurrence [1]. Factor V Leiden (FVL) and prothrombin G20210A (PT-G20210A) are genetic risk factors associated with twice the risk of recurrence in carriers compared to non-carriers [2]. To reduce the risk of recurrence, patients are often treated with anticoagulants [2, 3] which may cause bleeding as a side-effect, especially among carriers of gene variants e.g. CYP2C9 2*/3* and Vitamin K epoxide reductase complex 1 associated with twice the risk of bleeding compared to non-carriers [4].

Despite the known contribution of genes, guiding anticoagulation therapy based on genetic testing—prolonged anticoagulation for carriers of genes associated with higher risk of recurrence and genotype-guided dosing for carriers of genes associated with higher risk of bleeding—has not been routinely implemented in VTE management. This may be due to clinical guidelines that recommend not to test for genes associated with higher risk of recurrent VTE [2, 3, 5], especially among patients with transient risk related to oral contraception, major surgeries and chemotherapy. Genetic testing is currently recommended only for patients with persistent risk factors such as those related to age or lifestyle, who have first-degree relatives with VTE [2, 3], and the decision to initiate or to stop anticoagulation does not rely on the test finding alone [2].

Nevertheless, with the discovery of more genes associated with recurrent VTE [6, 7] and the increasing accessibility of genetic test to clinicians and patients [8], whether it is cost-effective to incorporate these tests in clinical settings should be examined and discussed. The extent and the quality of the existing economic evidence can be ascertained via a systematic review.

The most recent review of economic evaluations of genetic-guided pharmacotherapy for VTE, published in 2012 [9] found seven studies based on models, but had several gaps. Firstly, only four studies identified focused on patients with persistent risk factors, for whom selective testing based on family history is currently recommended [2, 3]. These modelling studies rarely involved clinicians in the model development and hence likely did not reflect actual clinical contexts. In addition, the review did not elaborate the clinical contexts being considered and the consequences of genetic testing accounted for, to allow clinicians to assess whether the findings would be relevant for their settings. The review did not examine variables that may affect the cost-effectiveness of genetic testing to inform the development of future models. Finally, the review appraised the reporting but not the methodological quality.

To address these gaps, our study identifies and analyses economic evaluations of pharmacogenetic-guided pharmacotherapy in patients with VTE to present (1) details on the provision of interventions and comparators to reflect the clinical contexts, (2) which consequences of genetic testing were accounted for, (3) the model variables that may influence cost-effectiveness and (4) the gaps in both reporting and methodological qualities. By addressing these gaps, clinicians and policy makers interested in implementing pharmacogenetic-guided pharmacotherapy in patients with VTE would be better informed of the strengths and weaknesses of existing economic evidence and can identify future research and policy measures to support the implementation.

## Methods

This study is part of a larger systematic review aiming to assess the economic evidence of genetic-guided pharmacotherapy in patients with CVD (prospectively registered on PROSPERO ID: CRD42019144579 [10]).

### Search strategies and study selection

The methodological details are available on the register and in Appendix 1, guided by the Preferred Reporting Items for Systematic Reviews and Meta-Analyses statement (Appendix 2) [11]. Briefly, we systematically searched three general bibliographic databases (Medline, Embase, Web of Science Core Collection) and three subject-specific bibliographic databases (Econlit, NHS Economic Evaluation Database, Health Technology Assessment) from inception until 29 June 2020 (Appendix 3). The database searches were supplemented [12] by searching the websites of four health technology assessment (HTA) agencies (UK NICE; Canadian CADTH; French HAS; Dutch ZonMw). We also searched reference lists of included articles and systematic or narrative review articles, and citations of included articles on Scopus. The titles and abstracts were double screened for potential eligibility after duplicates were removed, with disagreements resolved through discussion. Articles were included if they: reported a full economic evaluation based on models, trials or quasi-observational studies; and focused on genetic testing followed by pharmacotherapy for patients with VTE, where the VTE was not due to transient risk factors. Articles were excluded if they considered hypothetical genetic tests, used animals, or were review articles, study protocols, editorials, commentaries, opinions, conference abstracts or letters.

### Data extraction

We extracted author details, study design, sample characteristics, details on provision of genetic testing and comparator interventions, costs, outcome measures, analyses performed, the model variables and the base-case conclusion on cost-effectiveness based on the local willingness-to-pay (WTP) threshold. Base-case is the scenario which operationalises the best available estimates of the model variables as identified by the authors of the studies.

The two sections on details on the provision of genetic testing and its comparators were operationalized from the Template for Intervention Description and Replication [13] (Appendix 4).

In extracting the model variables, we indicated whether the variables were tested in one-way deterministic sensitivity analyses (DSA) and which, within the range tested, were influential in changing the base-case conclusion. One-way DSA is a simple sensitivity analysis in economic evaluations where a point estimate of a model variable is varied while keeping the others constant, to examine whether the variable could change the base-case conclusion (e.g. from being cost-effective to not cost-effective).

To assess the range of consequences of genetic testing captured by the studies, the impact inventory recommended by the Second Panel on Cost-effectiveness in Health and Medicine was used [14, 15]. The impact inventory is a list of 21 consequences an intervention may have inside and outside of healthcare sector [14, 15].

### Quality assessment

Reporting quality was assessed using the checklist developed by the Second Panel (Appendix 5, with guidelines quoted from [14, 15] to support judgement). Each of the 47 items was rated “Yes”, “No”, “Partial” or “Not Applicable”.

Methodological quality was assessed using the extended version of Consensus Health Economic Criteria List (CHEC-Extended) [16, 17]. Each of the 20 items, as recommended [16, 17] was rated “Yes/rather yes”, “No/rather no” or “Unclear”.

Double data extraction and double quality ratings were undertaken independently, with reference to a third reviewer on the interpretation of items for the first two papers and when any discrepancies could not be resolved for the remaining eight papers.

### Data analyses and presentation

To provide an overview, the study design and sample characteristics are presented as counts and percentages. Details of the interventions and comparators are visualised in a network diagram. The consequences of genetic testing accounted for in the studies are presented by study perspective, as recommended [14, 15].

A narrative synthesis of the economic evaluation findings are provided in a permutation matrix [18]. This 3 × 3 matrix presents each intervention in terms of whether its relative costs and relative effects are better, no different or worse from the comparator in the base-case. Interventions that appear in the bottom left of the matrix are less costly and more effective than the comparators and hence would be favoured for adoption in clinical practice. In contrast, interventions that appear in the top right are more costly and less effective than the comparators and hence would be rejected. Interventions that appear elsewhere in the matrix would require trading off costs and benefits, and comparison with a WTP threshold value prior to an adoption decision.

To examine the model variables influential in changing the base-case conclusion of the economic evaluations, we first organised the model variables into four categories: effectiveness, epidemiology, cost and utility. The epidemiological and the cost variables were subcategorised according to the framework of economic evaluations of genetic testing [19] and the cost categories in a related systematic review [20], respectively. Based on these categories and subcategories, we presented the number of model variables reported, tested in one-way DSA, with findings reported and were influential.

To examine the reporting and methodological quality of the included studies, we presented the percentage of items with each rating.

## Results

### Study inclusion

**Fig. 1:**
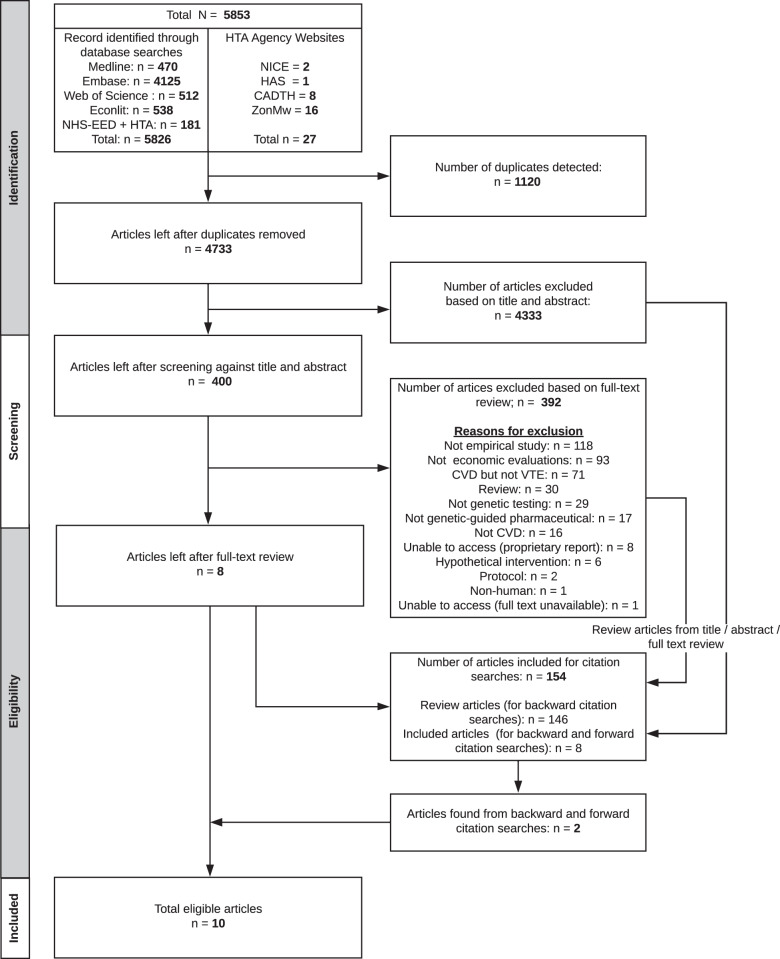
PRISMA flow chart. The flow chart indicates the flow of study selection, from searching the bibliographic databases and the websites of health technology assessment (HTA) agencies, to study screening and the inclusion of 10 eligible articles.

Of 5853 articles identified, 4733 were unique. From the unique articles, 4333 were removed after title and abstract screens and a further 392 after full-text screens. The top three reasons for exclusion from full-text screens were: not empirical study (e.g. editorials), not economic evaluation and economic evaluations on CVDs other than VTE. Citation searches of included papers identified two additional papers, resulting in a total of ten papers (Fig. 1).

### Study characteristics

**Fig. 2:**
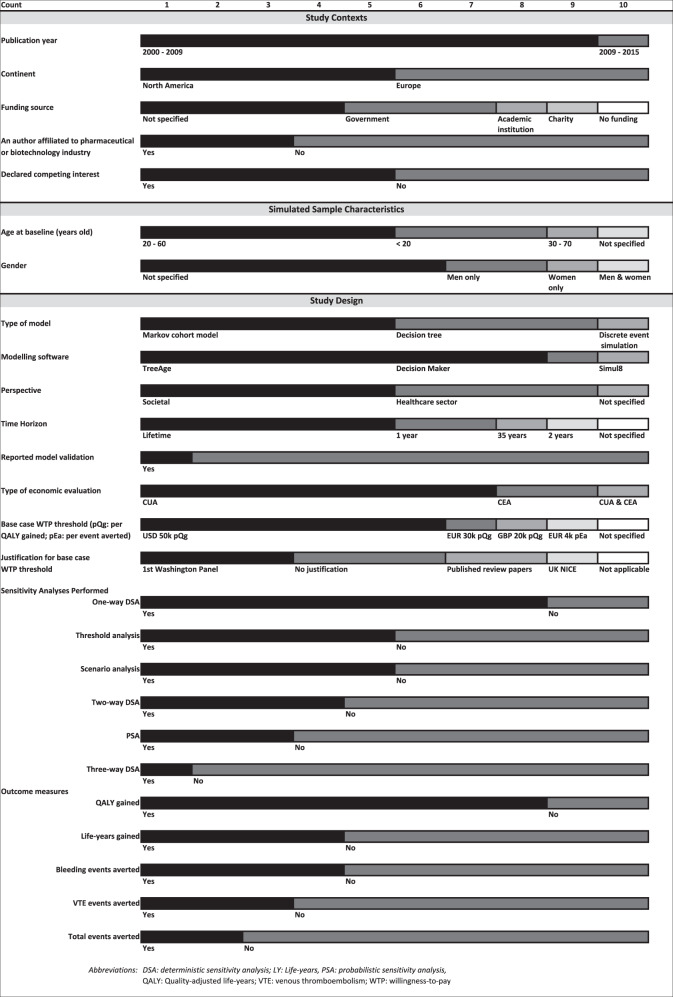
Characteristics of included studies. The horizontal bar charts represent the number of studies with each characteristic (study context, simulated sample characteristic and study design).

Figure 2 summarises characteristics of studies (see Appendix 6 for details). Most studies were published between 2009 and 2015, and were equally distributed between North America [21–25] and Europe [26–30].

All studies used models to assess the costs and the effects of genetic-guided pharmacotherapy. Half declared adopting a societal perspective; four [21, 25–27] assessed costs and effects over a lifetime and one [22] over 2 years. Only one study [29] reported its model development process and face validation with clinical experts.

Seven studies performed a cost-utility analyses (CUA), two a cost-effectiveness analyses (CEA) and one both a CUA and CEA. Most studies [21, 25–27, 29, 30] simulated patients aged 20–60 years at baseline; one study [22] simulated paediatric patients (2–18 years old); one study [30] simulated multiple patient subgroups aged 30–70 years. Contrary to the clear reporting of age, most studies did not explicitly report the gender of their simulated patients. No study reported the family history or CVD risk factors of their simulated patients.

### Interventions and comparators

**Fig. 3:**
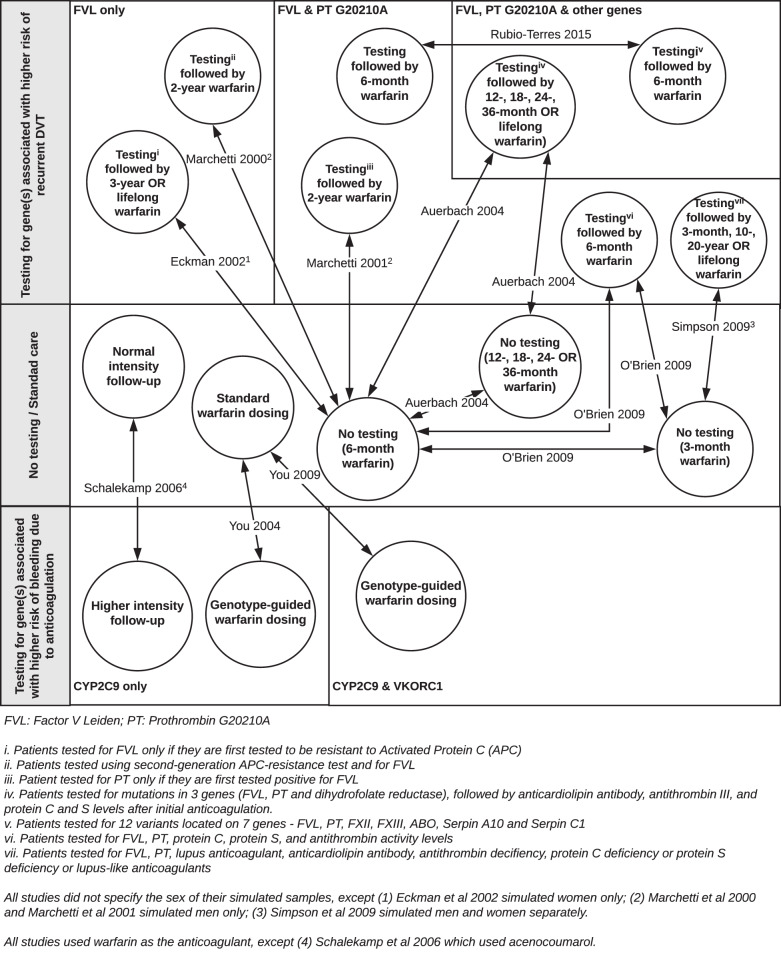
Overview of interventions and comparators. The network diagram summarises the types of genetic-guided pharmacotherapy and the comparators for the 10 included studies.

The interventions and comparators were heterogeneous (Fig. 3). Interventions differed in the type of genes tested, the number of genes tested and, among eight studies that tested multiple genes (with or without anticoagulant biomarkers, e.g. activated protein C), the test sequence. On the types of genes, seven studies [21, 22, 25–27, 29, 30] examined testing of genes associated with higher risk of recurrent VTE, whereas three [23, 24, 28] examined testing of genes associated with higher risk of bleeding with anticoagulation. Patients tested positive with the former were treated with prolonged anticoagulation therapy (≥6 months) whereas patients tested positive with the latter had genotype-guided dosing [23, 24] or higher intensity of follow-up [28]. In offering the test or deciding the duration of anticoagulation, no consideration of family history or risk factors other than the test finding was mentioned. On the number of genes tested, earlier studies [21, 24, 26, 28] tested only a single gene, later studies tested two [22, 23, 27, 30] or three [25] genes and the latest study [29] tested seven genes. On the test sequence, four studies offered sequential testing–anticoagulant biomarker before gene testing [21, 26], gene testing before anticoagulant biomarker [25] or one gene after another [27]; four studies offered simultaneous testing of multiple genes [23, 29] or genes with biomarkers [22, 30].

The comparators differed with regards to the interventions examined. Among studies that tested genes associated with higher risk of recurrent VTE, the comparators were mostly no-testing, with duration of anticoagulation (3 months to 3 years) shorter than that for patients tested positive. Only in one study [29], instead of no-testing, the comparator was testing fewer genes; this was the only study where the duration of anticoagulants did not differ between intervention and comparator. Meanwhile, for studies that tested genes associated with higher risk of bleeding, their comparators were standard warfarin dosing [23, 24] or normal intensity of follow-up [28].

All included studies used warfarin as the anticoagulant except one [28] that used acenocoumarol. None of the studies explicitly reported the setting in which the testing took place—two [24, 28] mentioned “anticoagulation clinics” without specifying whether these clinics were located in primary or secondary care settings; one study [30] mentioned testing was available in specialist laboratories or hospitals, but did not state which case was modelled. No study reported the testing procedures (e.g. blood, saliva) or the providers involved (e.g. nurse, lab technician). All studies assumed 100% uptake of genetic testing and only two studies [26, 29] considered less than perfect adherence to anticoagulation.

### Consequences of genetic testing accounted for

Based on the impact inventory (Appendix 7), all studies accounted for >1 consequence of genetic testing within the formal healthcare sector. The three most common consequences were health-related quality-of-life (*n* = 8), future-related medical costs (*n* = 6) and other health effects (*n* = 5). No study accounted for costs incurred by third-party payers, patients out-of-pocket spending or future unrelated medical costs. Of five studies that declared societal perspective, only three accounted for consequences outside formal healthcare sector, specifically labour marketing earnings loss [22, 26, 27] and transportation costs for physician visits and anticoagulation monitoring [22].

### Economic evaluation findings

**Fig. 4:**
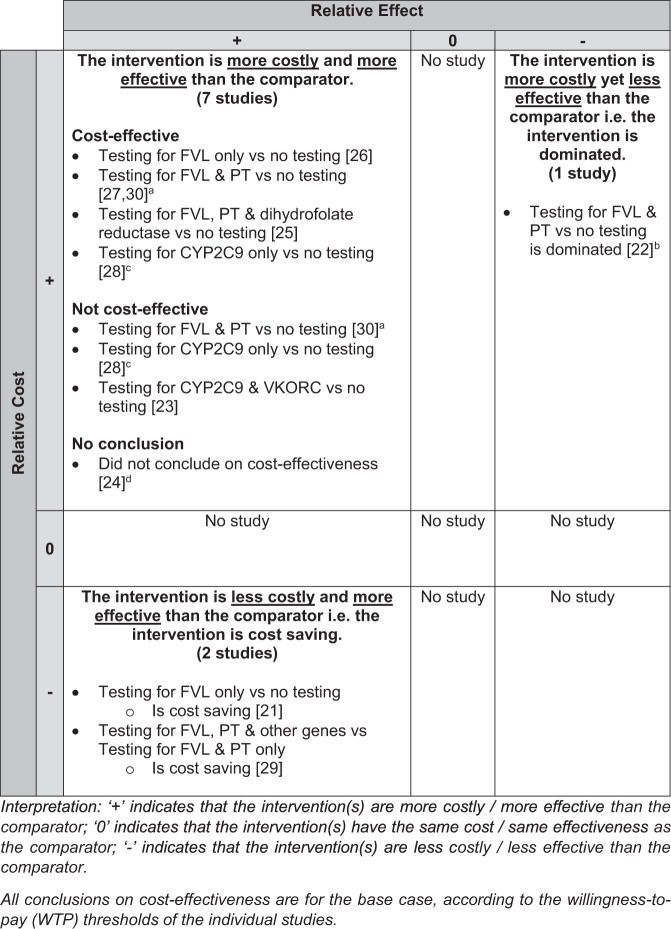
Findings of economic evaluations. The 3 × 3 matrix presents each intervention in terms of whether its relative costs and relative effects are higher/better (+), no different (0) or lower/worse (−) from the comparator in the base-case.

The base-case conclusions were distributed across three corners of the matrix (Fig. 4). The robustness of some base-case conclusions was further examined in different scenarios (scenario analyses) or in repeated simulations with varied model variables to estimate the probability of the intervention being cost-effective (probabilistic sensitivity analyses (PSA)).

Of the seven studies testing for genes associated with higher risk of recurrent VTE, six were at the bottom left or the top left of the matrix, concluding that at base-case, testing may be cost-saving, cost-effective or not cost-effective. Among the two that concluded cost-saving, one [21] showed via scenario analyses that genetic testing compared to no-testing remained cost-saving even with different assumptions on the persistence of recurrent VTE risk; while the other [29] demonstrated in PSA that testing for seven genes had a 100% probability of cost-saving compared to testing for two genes. One study [30] simulated multiple patient subgroups and found that in patients with PE, testing was cost-effective regardless of age and gender whereas in patients with DVT, testing was cost-effective in men <70 and women <50 years old. In the PSA of this study, however, testing was cost-effective only in 30–60% simulations among the subgroups. Three other studies also found testing cost-effective, with similar incremental cost-effectiveness ratios (USD11,100–13,624 per QALY gained [25–27]) over a patient’s lifetime despite testing different number of genes. None, however, examined the robustness of their base-case conclusion in scenario analyses or PSA. Meanwhile, at the top right corner, testing was dominated by no-testing in the only study that simulated paediatric patients [22].

All three studies that analysed testing for genes associated with higher risk of bleeding were in the top left corner. One [23] concluded that testing was not cost-effective and found, using PSA, a low probability of testing being cost-effective (19.1%) or cost-saving (18.7%). The other study had two base-cases—testing was cost-effective in the base-case with higher prevalence but not cost-effective in the base-case with lower prevalence of high-risk gene variants. However, it did not further examine the robustness of its base-case conclusions in PSA. The last study [24], without a WTP threshold, did not conclude on cost-effectiveness.

### Influential model variables

A total of 309 model variables were reported, approximately half were epidemiological variables, followed by cost, utility and effectiveness or relative effectiveness of interventions.

Overall, 53% variables (164/309) were tested in one-way DSA. Of 134 variables with findings reported, 22 were influential in changing the base-case conclusion, with the most frequent (5/22) being effectiveness or relative effectiveness of the intervention. However, these effectiveness estimates were not based on any trials of genetic testing in patients with VTE. Instead, they were based on randomised trials of genetic testing in non-VTE patients (23); observational studies and/or a randomised trial of prolonged warfarin in preventing recurrent VTE [21, 26, 27]; or assumption without elaboration, for the reduction in out-of-range INR [28]. The other influential variables were the risk of bleeding or recurrent VTE (with or without high-risk gene variants), prevalence of high-risk gene variants, cost of genetic testing, cost of warfarin monitoring, cost of treatment of bleeding and utility while taking warfarin (Table 1).

**Table 1:** Model variables reported, tested and shown to change base-case conclusion on cost-effectiveness.

Type of model input	Number of model variables			
Variables	Reported	Tested in one-way DSA	Findings available	Changed conclusion
Effectiveness/relative effectiveness of intervention	13	10	7	5
Epidemiological Variables				
Assay characteristics	36	34	34	0
Prevalence of high-risk gene variant(s)	11	5	4	2
Prevalence of biomarkers^a^	3	0	0	0
Baseline/relative risk in those with high-risk gene variant(s)				
Recurrent VTE^b^	8	5	5	1
Bleeding^c^	3	2	1	1
Baseline/relative risk in those without high-risk gene variant(s)				
Recurrent VTE^a^	56	25	22	3
Bleeding^c^	42	21	14	4
VTE complications^d^	12	1	1	0
Other epidemiological variables^e^	6	6	4	1
Cost/resource use/unit price				
Genetic testing	11	8	7	2
Testing of biomarkers^f^	1	0	0	0
Anticoagulation monitoring^g^	16	7	4	1
Treatment of VTE	23	12	10	0
Treatment of VTE complications^d^	12	0	0	0
Treatment of bleeding	23	11	8	1
Death	1	1	1	0
Utility				
Anticoagulation	6	4	3	1
VTE	7	3	2	0
VTE complications^d^	6	1	1	0
Bleeding	8	5	3	0
No event/off-treatment	2	2	2	0
Other utility^h^	3	1	1	0
Total variables	309	164	134	22

^a^Prevalence of lupus anticoagulant, anticardiolipin antibody and antithrombin, protein C or protein S deficiency.

^b^Venous thromboembolism and deaths due to venous thromboembolism.

^c^Haemorrhage and deaths due to haemorrhage.

^d^Includes non-haemorrhagic stroke and post‐thrombophlebitis syndrome.

^e^Compliance to anticoagulation and probability of deaths.

^f^Testing for Activated Protein C (in Eckman et al. 2002 [21], a patient must first be shown to be sensitive to Activated Protein C to be eligible for genetic testing).

^g^Cost of anticoagulation medication and/or monitoring.

^h^Utility for short stay in hospitals and utility for death.

### Reporting quality

**Fig. 5:**
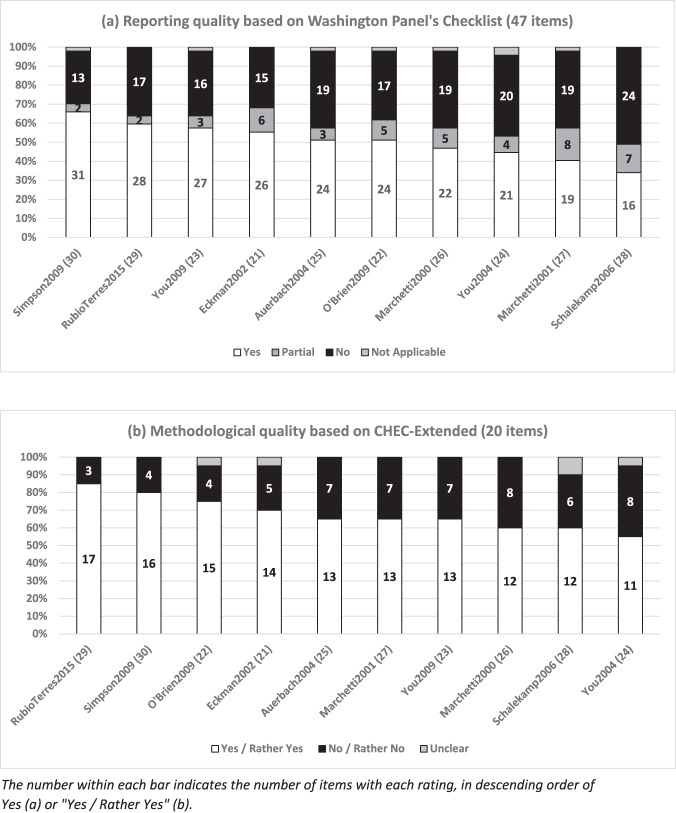
The vertical bar charts summarise the ratings for reporting and methodological quality respectively. The y-axes of both charts represent the percentage of items whereas the numbers within the bars indicate the number of items with each rating. **a** Reporting and **b** methodological quality ratings.

Overall, 34–66% of the 47 items in Second Panel’s reporting checklist were rated “Yes” (Fig. 5a and Appendix 8). Ten items were reported by all studies (e.g. type of analysis, software used) whereas six were not reported by any study (e.g. intervention details, results of model validation). Other items less commonly reported were methods for obtaining data, critique of data quality and discussion of ethical implications of genetic testing.

### Methodological quality

Overall, 55–85% of the 20 items in CHEC-Extended were rated “Yes/rather yes” (Fig. 5b and Appendix 9). Six items were reported by all studies (e.g. economic study design) whereas one item (i.e. ethical and distributional issues) was not discussed appropriately in any study. Other items less reported were structural assumptions, validation methods and discussion of generalisability.

## Discussion

Our review systematically examined economic evaluations of genetic-guided pharmacotherapy in patients with VTE driven by persistent risk factors. We found ten studies, six more than the previous review [9]. These studies, modelling a heterogeneous network of interventions and comparators, examined genetic testing followed by prolonged anticoagulation (for genes associated with higher risk of recurrent VTE) or genetic testing followed by genotype-guided dosing or higher follow-up intensity (for genes associated with higher risk of bleeding). All studies were models, mostly accounted for consequences of genetic testing within formal healthcare sector. Gaps in reporting and methodological qualities were apparent, with studies lacking details on the delivery of interventions and comparators as well as on model development and validation. Results on cost-effectiveness were mixed, ranging across being cost-saving, cost-effective, to being too costly for existing cost-effectiveness thresholds and dominated by comparators. The heterogeneity of interventions and mixed findings means it is not yet clear which type of genetic testing is cost-effective or cost-saving.

As the studies were published before recent clinical guidelines [2, 3], it is not surprising that none modelled the currently recommended test strategies [2]—to offer testing only to those with strong family history and to decide on the initiation or the continuation of anticoagulation therapy on the basis of other risk factors as well as the test finding. None examined testing for extended list of genes recently found to be associated with higher risk of recurrent VTE for which a multiple-gene panel is available [6, 7]. In addition, none explored testing both sets of genes, to reduce the risk of recurrent VTE as well as bleeding. These are potential test strategies that future economic evaluations could examine.

Of the list of 22 model variables found to change findings on cost-effectiveness, the most frequent variable was effectiveness or relative effectiveness of the intervention. The quality of data used for effectiveness was not high, however, as they lack directly relevant trial data in patients with VTE. Existing trials [31] and meta-analysis of trials [32, 33], although some do not demonstrate benefits of genetic-guided pharmacotherapy of anticoagulant, are not specific to patients with VTE. While some may consider that the incremental risk attributable to currently known genes (FVL and PT-G20210A) are not high enough to change treatment decision [8], this may need to be revisited when a new gene or a panel of genes associated with risk higher than currently known ones are discovered. New technologies suggest this is imminent [6, 7]. In this case, our list of variables could inform the design of data collection forms for trials as well as the cost-effectiveness models.

The list of variables has two shortcomings, nevertheless. First, it is likely incomplete. For example, three studies [22, 26, 27] accounted for loss of earnings due to VTE but did not report the values. Secondly, details on costs were lacking. For example, only one study specified whether the cost of genetic tests included consumables or overheads [24], with the rest ambiguous; poor reporting of the setting in which the genetic testing was delivered made it challenging to account for the variability in costs between different delivery settings. Finally, the uptake of genetic testing was notably absent since all studies assumed perfect uptake. This is unlikely to match reality, especially if positive results cause stress and anxiety [34]. Future evaluations should account for test uptake.

The impact inventory indicates that some relevant consequences related to genetic testing or VTE were not accounted for (e.g. patient time). Accounting for these consequences may make testing more cost-effective if pharmacotherapy following testing can prevent or reduce them. One consequence of genetic testing not listed in the impact inventory is the psychological impact of knowing the genetic test findings (the “value of knowing” [35]). The value of knowing may also extend to family members (of those tested positive with genes associated with higher risk of recurrent VTE) who receive cascade testing, although the clinical benefit of cascade testing for family members is still unclear [2, 3]. Neither the value of knowing nor cascade testing was examined by the included studies, hence how these may have changed the findings is also unknown and can be explored in future studies.

Our review has several limitations. First, it excluded patients with transient risk factors of VTE, e.g. those taking contraceptives or undergoing major surgeries. However, excluding them allow us to provide a focused summary of evidence for those with persistent risk factors. Second, our findings may not be generalisable to settings where direct oral anticoagulants is used [36, 37], as no included studies used them as the anticoagulant. Third, as all studies examined VTE as a whole, it remains unknown whether there is any VTE subtype for which genetic-guided pharmacotherapy may be more favourable. Next, in the absence of recommended rating options, we used our own operationalization of the Second Panel’s reporting checklist based on the Panel’s publications [14, 15]. We shared the guidance we compiled to support our judgement in Appendix 5. Fourth, while we differentiated reporting and methodological quality by using two different checklists, both checklists ultimately relied on reporting. This may explain why included studies with better reporting quality also appeared to have better methodological quality.

Despite the limitations, our review has strengths and contributions to the literature. First, compared to the previous review that searched three databases [9], we searched six databases and the websites of four HTA agencies, with forward and backward citation searches. Second, our structured approach in examining the interventions and comparators revealed that the interventions and the comparators were often insufficiently described to provide clinical contexts being considered, and that most economic evaluations only accounted for consequences within the healthcare sector. These, in addition to the list of influential variables we presented would inform future economic evaluations on the topic.

## Conclusion

Our review found ten studies, all model-based, of genetic-guided pharmacotherapy for patients with VTE. With the heterogeneous interventions and comparators, gaps in quality of reporting and wide range in findings, it was not possible to pinpoint which type of genetic testing would be cost-effective or cost-saving. Several possible test strategies based on the guidelines and the literature were also notably missing. This includes testing based on family history and offering anticoagulation therapy in consideration of other risk factors in addition to test finding, testing genes recently discovered or testing both genes associated with higher risk of recurrent VTE and those associated with higher risk of bleeding. Besides examining these strategies and testing key model variables for decision uncertainty, future economic evaluations should improve the reporting of interventions, comparators and methodological details, to advise clinicians whether and when to adopt genetic-guided pharmacotherapy, especially with the discoveries of new high-risk genes and increasing availability of genetic testing.

## Supplementary information

Appendices 1–9
