# Supplementary material for: Genetic-guided pharmacotherapy for venous thromboembolism: a systematic and critical review of economic evaluations
Source: Pharmacogenomics J. 2021 Jun 15;21(6):625–37. doi: 10.1038/s41397-021-00243-7 (PMC8602036; doi:10.1038/s41397-021-00243-7)
Supplement: Supplementary file 1 — Appendices 1–9 [file 41397_2021_243_MOESM1_ESM.docx]

**Genetic-guided pharmacotherapy for venous thromboembolism: A systematic and critical review of economic evaluations**

*Lim KK, *Koleva-Kolarova R, (Co-first authors) Chowienczyk P, Wolfe C, Fox-Rushby J

Appendix 1 Methodological details

Appendix 2 PRISMA checklist

Appendix 3 Search strategies

Appendix 4 Operationalization of the Template for Intervention Description and Replication (TIDieR) to collect details pertaining delivery of genetic testing and comparators.

Appendix 5 Operationalization of the Second Washington Panel’s reporting checklist

Appendix 6 One-page summaries of included studies

Appendix 7 Impact of genetic-guided pharmacotherapy accounted for

Appendix 8 Reporting quality ratings of included studies in full, based on the Second Washington Panel’s reporting checklist

Appendix 9 Methodological quality ratings of included studies in full, based on CHEC-Extended checklist

# Appendix 1 Detailed description of methodology

The Preferred Reporting Items for Systematic Reviews and Meta-Analyses (PRISMA) statement was used to guide the design of the search strategies and the screening of articles for the systematic review as well as the analysis and the reporting of the findings (Appendix 2).

## Search strategies

We systematically searched three general bibliographic databases (Medline, Embase, Web of Science Core Collection) and three subject-specific bibliographic databases (Econlit, NHS Economic Evaluation Database, Health Technology Assessment) from inception until 29 June 2020. We developed the search strategies first on Medline using a combination of Medical Subject Headings (MeSH) and free texts, guided by an experienced librarian and in consultation with our clinical co-authors before adapting them to other databases. Our search strategies (Appendix 3) covered three concepts – economic evaluation, pharmacogenetics and CVD. VTE and its synonyms were included within the search terms for CVD.

The database searches were supplemented by searching the websites of four health technology assessment (HTA) agencies (National Institute for Health and Care Excellence, NICE; Canadian Agency for Drugs and Technologies in health, CADTH; French National Authority for Health, HAS; Netherlands Organisation for Health Research and Development, ZonMw). We also searched reference lists of included articles and systematic or narrative review articles, and citations of included articles on Scopus.

## Study selection

Duplicate articles were removed in Microsoft Excel (Microsoft, Seattle, WA) and EndNote (Clarivate Analytics, Philadelphia, USA) before screening. Titles and abstracts were double screened for potential eligibility followed by full texts of shortlisted articles. Any disagreement on eligibility was resolved through discussion between the two screeners or when necessary, with a third screener.

Articles were included if they: reported a full economic evaluation (i.e. reporting costs and effects of at least two alternatives) based on models, trials or quasi-observational studies; and focused on genetic testing followed by pharmacotherapy for patients with VTE, where the VTE was not due to transient risk factors. No limitations were applied on the age, gender or ethnic background of the study populations or the setting where genetic testing took place.

Articles were excluded if they considered hypothetical genetic tests, used animals, or were review articles, study protocols, editorials, commentaries, opinions, conference abstracts or letters.

## Data extraction

We used a piloted data extraction form comprising nine sections: author details, study design, sample characteristics, details on provision of genetic testing and comparator interventions, costs, outcome measures, analyses performed, the model variables and the base-case conclusion on cost-effectiveness based on the local willingness-to-pay (WTP) threshold. Base case is defined as the scenario which operationalises the best available estimates of the model variables as identified by the authors of the studies.

The two sections on details on the provision of genetic testing and its comparators were operationalized from the Template for Intervention Description and Replication (TIDieR) [1], to extract data on the genes, the setting in which genetic testing took place, the mode of delivery, the providers involved and how anticoagulation therapy changed as a result of test finding (Appendix 4).

In extracting the model variables, we indicated whether variables were tested in one-way deterministic sensitivity analyses (DSA) and which, within the range tested, were influential in changing the base-case conclusion. One-way DSA is a simple sensitivity analysis in economic evaluations that accounts for variability and uncertainty where a point estimate of a model variable is varied while keeping the others constant, to examine whether the variable could change the base-case conclusion (e.g. from being cost-effective to not cost-effective).

To assess the range of consequences of genetic testing captured by the studies, the impact inventory recommended by the Second Panel on Cost-effectiveness in Health and Medicine was used [2, 3]. The impact inventory is a list of 21 consequences an intervention may have inside and outside of healthcare sector and is based on the perspective of an economic evaluation. Adopting a healthcare sector perspective, an economic evaluation may account for formal healthcare costs including current and future costs incurred by government, third-party payers or patients, whereas adopting a societal perspective, all costs and effects would be included regardless of who incurs them [2, 3]..

## Quality assessment

We assessed the included studies for reporting and methodological quality. Reporting quality was operationalised from the checklist developed by the Second Panel [2, 3], which delineates the reporting of an economic evaluation in 47 items (Appendix 5, with guidelines quoted from [2, 3] to support judgement). Each item, in the absence of a recommended rating options by the Panel, was rated “Yes” (when an item was reported), “No” (when an item was not reported), “Partial” (when an item consists of multiple components and some components were reported), or “Not Applicable”.

Methodological quality was operationalised from the extended version of Consensus Health Economic Criteria List (CHEC-Extended) [4, 5]. CHEC-Extended, commonly used in systematic reviews of economic evaluations [6], contains 20 items on the appropriateness in the conduct of an economic evaluation. These include, for example, the structural assumptions and the validation methods of the model, whether a study accounts for important and relevant costs, and whether a study performs appropriate incremental analysis. Each item, as recommended by the checklist developers [4, 5], was rated “Yes / rather yes”, “No / rather no”, or “Unclear”.

Double data extraction and double quality ratings were undertaken independently, with reference to a third reviewer on the interpretation of items for the first two papers and where any discrepancies could not be resolved with discussion for the remaining eight papers.

## Data analyses and presentation

To provide an overview, the study design and sample characteristics are presented as counts and percentages. Details of the interventions and comparators are visualized in a network diagram. The consequences of genetic testing accounted for in the studies are presented by study perspective, as recommended [2, 3].

Due to the heterogeneity in study characteristics, interventions, comparators and the consequences of genetic testing accounted for, meta-analysis of the findings was not feasible. Thus, a narrative synthesis of the economic evaluation findings are provided in a permutation matrix [7]. This 3x3 matrix presents each intervention in terms of whether its relative costs and relative effects are better, worse or no different from the comparator in the base-case. Interventions that appear in the bottom left of the matrix are less costly and more effective than the comparators and hence would be favoured for adoption in clinical practice. In contrast, interventions that appear in the top right are more costly and less effective than the comparators and hence would be rejected. Interventions that appear elsewhere in the matrix would require trading off costs and benefits, and comparison with a WTP threshold value prior to an adoption decision.

To examine the model variables influential in changing the base-case conclusion of the economic evaluations, we first organized the model variables into four categories: effectiveness, epidemiology, cost and utility. The epidemiological variables were subcategorized, adapting from the framework of economic evaluations of genetic testing [8], into assay characteristics (e.g. test sensitivity or specificity), prevalence of gene variants, prevalence of other biomarkers, baseline or relative risk among those with or without the gene variants, or other variables. Cost variables were subcategorized, adapting from the cost categories in a systematic review of related topic [9], into cost of genetic testing, testing of other biomarkers, warfarin monitoring, treatment of VTE, treatment of VTE complications (e.g. stroke or post-thrombophlebitis syndrome), treatment of bleeding or death. Similarly, utility variables related to quality of life were subcategorized as utility while under warfarin, VTE, VTE complications, bleeding, no-event or off-treatment, or others (e.g. utility for short-stay in hospitals not specific to a disease). Based on these categories and subcategories, we presented the number of model variables reported, number of variables tested in one-way DSA, number of variables with findings available for extraction and number of variables that were influential.

To examine the reporting and methodological quality of the included studies, in the absence of a consensus on what constitutes good or poor quality, we presented the percentage of items with each rating.

To provide a succinct view of each study, a one-page summary is provided for each (see Appendix 6)

# Appendix 2 PRISMA Checklist

| **Section/topic** | **#** | **Checklist item** | **Reported on page #** |  |  |
| --- | --- | --- | --- | --- | --- |
| **TITLE** | | |  |  |  |
| Title | 1 | Identify the report as a systematic review, meta-analysis, or both. | 1 & 2 |  |  |
| **ABSTRACT** | | |  |  |  |
| Structured summary | 2 | Provide a structured summary including, as applicable: background; objectives; data sources; study eligibility criteria, participants, and interventions; study appraisal and synthesis methods; results; limitations; conclusions and implications of key findings; systematic review registration number. | 4 |  |  |
| **INTRODUCTION** | | |  |  |  |
| Rationale | 3 | Describe the rationale for the review in the context of what is already known. | 5-6 |  |  |
| Objectives | 4 | Provide an explicit statement of questions being addressed with reference to participants, interventions, comparisons, outcomes, and study design (PICOS). | 6 |  |  |
| **METHODS** | | |  |  |  |
| Protocol and registration | 5 | Indicate if a review protocol exists, if and where it can be accessed (e.g., Web address), and, if available, provide registration information including registration number. | 7 |  |  |
| Eligibility criteria | 6 | Specify study characteristics (e.g., PICOS, length of follow-up) and report characteristics (e.g., years considered, language, publication status) used as criteria for eligibility, giving rationale. | 7-8 |  |  |
| Information sources | 7 | Describe all information sources (e.g., databases with dates of coverage, contact with study authors to identify additional studies) in the search and date last searched. | 7 |  |  |
| Search | 8 | Present full electronic search strategy for at least one database, including any limits used, such that it could be repeated. | 7, Appendix 3 |  |  |
| Study selection | 9 | State the process for selecting studies (i.e., screening, eligibility, included in systematic review, and, if applicable, included in the meta-analysis). | 7 |  |  |
| Data collection process | 10 | Describe method of data extraction from reports (e.g., piloted forms, independently, in duplicate) and any processes for obtaining and confirming data from investigators. | 7-8 |  |  |
| Data items | 11 | List and define all variables for which data were sought (e.g., PICOS, funding sources) and any assumptions and simplifications made. | 7-8 |  |  |
| Risk of bias in individual studies | 12 | Describe methods used for assessing risk of bias of individual studies (including specification of whether this was done at the study or outcome level), and how this information is to be used in any data synthesis. | 8 |  |  |
| Summary measures | 13 | State the principal summary measures (e.g., risk ratio, difference in means). | NA |  |  |
| Synthesis of results | 14 | Describe the methods of handling data and combining results of studies, if done, including measures of consistency (e.g., I^2^) for each meta-analysis. | 8-9 |  |  |

Page 1 of 2

| **Section/topic** | **#** | **Checklist item** | **Reported on page #** |  |  |
| --- | --- | --- | --- | --- | --- |
| Risk of bias across studies | 15 | Specify any assessment of risk of bias that may affect the cumulative evidence (e.g., publication bias, selective reporting within studies). | NA |  |  |
| Additional analyses | 16 | Describe methods of additional analyses (e.g., sensitivity or subgroup analyses, meta-regression), if done, indicating which were pre-specified. | NA |  |  |
| **RESULTS** | | |  |  |  |
| Study selection | 17 | Give numbers of studies screened, assessed for eligibility, and included in the review, with reasons for exclusions at each stage, ideally with a flow diagram. | 10 |  |  |
| Study characteristics | 18 | For each study, present characteristics for which data were extracted (e.g., study size, PICOS, follow-up period) and provide the citations. | 10-11, Figure 2 |  |  |
| Risk of bias within studies | 19 | Present data on risk of bias of each study and, if available, any outcome level assessment (see item 12). | 13, Figure 5, Appendix 8-9 |  |  |
| Results of individual studies | 20 | For all outcomes considered (benefits or harms), present, for each study: (a) simple summary data for each intervention group (b) effect estimates and confidence intervals, ideally with a forest plot. | 11-12 |  |  |
| Synthesis of results | 21 | Present results of each meta-analysis done, including confidence intervals and measures of consistency. | 12 |  |  |
| Risk of bias across studies | 22 | Present results of any assessment of risk of bias across studies (see Item 15). | NA |  |  |
| Additional analysis | 23 | Give results of additional analyses, if done (e.g., sensitivity or subgroup analyses, meta-regression [see Item 16]). | NA |  |  |
| **DISCUSSION** | | |  |  |  |
| Summary of evidence | 24 | Summarize the main findings including the strength of evidence for each main outcome; consider their relevance to key groups (e.g., healthcare providers, users, and policy makers). | 14 |  |  |
| Limitations | 25 | Discuss limitations at study and outcome level (e.g., risk of bias), and at review-level (e.g., incomplete retrieval of identified research, reporting bias). | 15 |  |  |
| Conclusions | 26 | Provide a general interpretation of the results in the context of other evidence, and implications for future research. | 16 |  |  |
| **FUNDING** | | |  |  |  |
| Funding | 27 | Describe sources of funding for the systematic review and other support (e.g., supply of data); role of funders for the systematic review. | 1 |  |  |

*From:*  Moher D, Liberati A, Tetzlaff J, Altman DG, The PRISMA Group (2009). Preferred Reporting Items for Systematic Reviews and Meta-Analyses: The PRISMA Statement. PLoS Med 6(7): e1000097. doi:10.1371/journal.pmed1000097

For more information, visit: **www.prisma-statement.org**.

Page 2 of 2

# Appendix 3 Search strategies

**Medline via Ovid ®**

| **No.** | **Search** |
| --- | --- |
| 1 | (Cost Benefit Analys$ or CBA).mp. or exp Cost-Benefit Analysis/ |
| 2 | Cost$ analys$.mp. or exp "Costs and Cost Analysis"/ |
| 3 | (Cost effectiveness or cost-effectiveness or CEA or cost-utility analys$ or cost utility analys$ or CUA).mp. |
| 4 | Pharmacoeconomic$.mp. or exp Economics, Pharmaceutical/ |
| 5 | Econ$ Evaluat$.mp. |
| 6 | 1 or 2 or 3 or 4 or 5 |
| 7 | Pharmacogenetic$.mp. or exp PHARMACOGENETICS/ |
| 8 | Pharmacogenomic$.mp. |
| 9 | Precision Medicin$.mp. or exp Precision Medicine/ |
| 10 | Individuali?ed Medicin$.mp. |
| 11 | Individuali?ed treatment$.mp. |
| 12 | Personali?ed Medicin$.mp. |
| 13 | Personali?ed treatment$.mp. |
| 14 | Personali?ed therap*.mp. |
| 15 | (Genetic$ screen$ or Genetic Test$).mp. or exp Genetic Testing/ |
| 16 | Genotype$.mp. or exp GENOTYPE/ |
| 17 | genetic marker$.mp. or exp Genetic Markers/ |
| 18 | genomic marker$.mp. |
| 19 | exp Genes/ or exp Mutation/ or genetic analys$.mp. or exp Phenotype/ |
| 20 | exp Genetic Variation/ or exp Genomics/ |
| 21 | 7 or 8 or 9 or 10 or 11 or 12 or 13 or 14 or 15 or 16 or 17 or 18 or 19 or 20 |
| 22 | cardiovascular disease$.mp. or exp Cardiovascular Diseases/ |
| 23 | coronary arter$ disease$.mp. or exp Coronary Artery Disease/ |
| 24 | Coronary Arteriosclero$.mp. |
| 25 | Angina Pectoris.mp. or exp Angina Pectoris/ |
| 26 | Myocardial Infarct$.mp. or exp Myocardial Infarction/ |
| 27 | Myocardial Reperfusion.mp. or exp Myocardial Reperfusion/ |
| 28 | exp MYOCARDIUM/ or Myocardium.mp. |
| 29 | Heart Attack$.mp. |
| 30 | exp STROKE/ |
| 31 | Heart Failure$.mp. or exp Heart Failure/ |
| 32 | exp HYPERTENSION, PULMONARY/ or exp HYPERTENSION/ or exp HYPERTENSION, RENAL/ or exp WHITE COAT HYPERTENSION/ or Hypertensi$.mp. or exp HYPERTENSION, RENOVASCULAR/ or exp HYPERTENSION, PORTAL/ or exp ESSENTIAL HYPERTENSION/ or exp HYPERTENSION, MALIGNANT/ or exp MASKED HYPERTENSION/ or exp HYPERTENSION, PREGNANCY-INDUCED/ |
| 33 | high blood pressure.mp. |
| 34 | Rheumatic Heart Disease$.mp. or exp Rheumatic Heart Disease/ |
| 35 | exp DIABETIC CARDIOMYOPATHIES/ or exp CARDIOMYOPATHIES/ or Cardiomyopath$.mp. |
| 36 | Heart Valve Disease$.mp. or exp Heart Valve Diseases/ |
| 37 | Myocarditis.mp. or exp MYOCARDITIS/ |
| 38 | Aortic Aneurysm$.mp. or exp Aortic Aneurysm/ |
| 39 | Peripheral Arterial Disease$.mp. or exp Peripheral Vascular Diseases/ or exp Peripheral Arterial Disease/ or exp Coronary Disease/ or exp Arteriosclerosis/ or exp Cardiovascular Diseases/ |
| 40 | Venous Thrombo$.mp. or exp Venous Thrombosis/ |
| 41 | Acute rheumatic fever.mp. or exp Rheumatic Fever/ |
| 42 | pericarditis.mp. or exp PERICARDITIS/ |
| 43 | endocarditis.mp. or exp ENDOCARDITIS/ |
| 44 | exp Heart Diseases/ or chronic rheumatic heart disease$.mp. |
| 45 | isch?emic heart disease$.mp. or exp Myocardial Ischemia/ |
| 46 | cerebrovascular disease$.mp. or exp Cerebrovascular Disorders/ |
| 47 | phlebitis.mp. or exp PHLEBITIS/ |
| 48 | thrombophlebitis.mp. or exp THROMBOPHLEBITIS/ |
| 49 | hypotension.mp. or exp HYPOTENSION, ORTHOSTATIC/ or exp HYPOTENSION/ |
| 50 | Rheumatic chorea.mp. or exp Chorea/ |
| 51 | Coronary thrombo$.mp. or exp Coronary Thrombosis/ |
| 52 | exp EMBOLISM/ or embolism$.mp. or exp PULMONARY EMBOLISM/ |
| 53 | Atheroscleros?s.mp. or exp ATHEROSCLEROSIS/ |
| 54 | Mitral steno$.mp. or exp Mitral Valve Stenosis/ |
| 55 | Atrial Fibrillation/ |
| 56 | ((atria* or atrium or auricular) adj6 fibril*).tw, kf, ot. |
| 57 | AF.tw,kf. and (flutter or fibril?at* or arr?yth?m* or atrial or atrium or atria).mp. |
| 58 | ((recurr* or persistent* or long-dur* or long-stand* or longstand* or long-last* or longlast* or prolonged or continuing or chronic* or refractory or non-valv* or nonvalv* or nonparoxysm* or non-paroxysm*) adj3 AF).tw. |
| 59 | (LPAF or LSPAF or LSP-AF or PsAF or Ps-AF or R-AF or PerAF or Per-Af or CPAF).tw. |
| 60 | 22 or 23 or 24 or 25 or 26 or 27 or 28 or 29 or 30 or 31 or 32 or 33 or 34 or 35 or 36 or 37 or 38 or 39 or 40 or 41 or 42 or 43 or 44 or 45 or 46 or 47 or 48 or 49 or 50 or 51 or 52 or 53 or 54 or 55 or 56 or 57 or 58 or 59 |
| 61 | 6 and 21 and 60 |
| 62 | limit 61 to humans |

**Embase via Ovid ®**

| **No** | **Search** |
| --- | --- |
| 1 | (cost effectiveness or cost-effectiveness).mp. |
| 2 | exp "cost effectiveness analysis"/ |
| 3 | "cost benefit analys$".mp. |
| 4 | exp "cost benefit analysis"/ |
| 5 | "cost utility analys$".mp. |
| 6 | exp "cost utility analysis"/ |
| 7 | econ$ evaluat$.mp. or exp economic evaluation/ |
| 8 | 1 or 2 or 3 or 4 or 5 or 6 or 7 |
| 9 | Pharmacogenetic$.mp. or exp pharmacogenetics/ |
| 10 | Pharmacogenomic$.mp. or exp pharmacogenomics/ |
| 11 | Precision Medicin$.mp. or exp personalized medicine/ |
| 12 | Individuali?ed Medicin$.mp. |
| 13 | Individuali?ed treatment$.mp. |
| 14 | Personali?ed Medicin$.mp. |
| 15 | Personali?ed treatment$.mp. |
| 16 | Personali?ed therap$.mp. |
| 17 | exp genetic screening/ or Genetic$ screen$.mp. |
| 18 | exp genetic analysis/ or Genetic Test$.mp. |
| 19 | exp genotype/ or Genotype$.mp. |
| 20 | genetic marker$.mp. or exp genetic marker/ |
| 21 | exp marker/ or genomic marker$.mp. |
| 22 | 9 or 10 or 11 or 12 or 13 or 14 or 15 or 16 or 17 or 18 or 19 or 20 or 21 |
| 23 | cardiovascular disease$.mp. or exp cardiovascular disease/ |
| 24 | coronary arter$ disease$.mp. or exp coronary artery disease/ |
| 25 | Coronary Arteriosclero$.mp. or exp coronary artery atherosclerosis/ |
| 26 | Angina Pectoris.mp. or exp angina pectoris/ |
| 27 | Myocardial Infarct$.mp. or exp heart infarction/ |
| 28 | Myocardial Reperfusion.mp. or exp heart muscle reperfusion/ |
| 29 | Myocardium.mp. or exp cardiac muscle/ |
| 30 | Heart Attack$.mp. or exp heart infarction/ |
| 31 | stroke$.mp. or exp cerebrovascular accident/ |
| 32 | Heart Failure$.mp. or exp heart failure/ |
| 33 | exp hypoxia-induced pulmonary hypertension/ or exp persistent pulmonary hypertension/ or exp hereditary hypertension/ or exp resistant hypertension/ or exp deoxycorticosterone-salt induced hypertension/ or exp masked hypertension/ or exp portal hypertension/ or exp chronic thromboembolic pulmonary hypertension/ or exp maternal hypertension/ or exp borderline hypertension/ or exp intraabdominal hypertension/ or exp malignant hypertension/ or exp portopulmonary hypertension/ or hypertension.mp. or exp systolic hypertension/ or exp renovascular hypertension/ or exp experimental pulmonary hypertension/ or exp white coat hypertension/ or exp diabetic hypertension/ or exp idiopathic intracranial hypertension/ or exp intracranial hypertension/ or exp intraocular hypertension/ or exp orthostatic hypertension/ or exp hypertension/ or exp essential hypertension/ or exp pulmonary hypertension/ or exp monocrotaline-induced pulmonary hypertension/ or exp experimental hypertension/ |
| 34 | high blood pressure.mp. |
| 35 | exp hypertrophic cardiomyopathy/ or exp restrictive cardiomyopathy/ or exp congestive cardiomyopathy/ or exp hypertrophic obstructive cardiomyopathy/ or exp nonischemic cardiomyopathy/ or exp tachycardia induced cardiomyopathy/ or cardiomyopathy.mp. or exp ischemic cardiomyopathy/ or exp cardiomyopathy/ or exp diabetic cardiomyopathy/ or exp peripartum cardiomyopathy/ or exp takotsubo cardiomyopathy/ or exp familial hypertrophic cardiomyopathy/ |
| 36 | Heart Valve Disease$.mp. or exp valvular heart disease/ |
| 37 | Aortic Aneurysm$.mp. or exp abdominal aorta aneurysm/ or exp aneurysm rupture/ or exp aortic aneurysm/ or exp aorta rupture/ or exp aorta aneurysm/ |
| 38 | Peripheral Arterial Disease$.mp. or exp peripheral occlusive artery disease/ |
| 39 | Peripheral Vascular Disease$.mp. or exp peripheral vascular disease/ |
| 40 | Coronary Disease.mp. or exp coronary artery disease/ |
| 41 | Arteriosclero$.mp. or exp experimental arteriosclerosis/ or exp arteriosclerosis/ or exp atherosclerosis/ or exp ischemic heart disease/ or exp peripheral occlusive artery disease/ or exp coronary artery atherosclerosis/ |
| 42 | heart disease$.mp. |
| 43 | exp heart ventricle extrasystole/ or exp heart disease/ or exp congestive heart failure/ or chronic heart disease.mp. or exp coronary artery disease/ |
| 44 | isch?emic heart disease$.mp. or exp ischemic heart disease/ |
| 45 | cerebrovascular disease$.mp. or exp cerebrovascular disease/ |
| 46 | Coronary Thrombo$.mp. or exp coronary artery thrombosis/ |
| 47 | exp embolism prevention/ or exp paradoxical embolism/ or exp fat embolism/ or exp kidney artery embolism/ or exp artery embolism/ or embolism.mp. or exp gas embolism/ or exp cholesterol embolism/ or exp air embolism/ or exp lung embolism/ or exp vein embolism/ or exp embolism/ |
| 48 | Atherosclero$.mp. or exp atherosclerosis/ or exp coronary artery atherosclerosis/ or exp aortic atherosclerosis/ or exp carotid atherosclerosis/ or exp brain atherosclerosis/ or exp experimental atherosclerosis/ |
| 49 | mitral steno$.mp. or exp mitral valve stenosis/ |
| 50 | Atrial Fibrillation/ |
| 51 | ((atria* or atrium or auricular) adj6 fibril*).tw,kw,ot. |
| 52 | AF.tw,kw. and (flutter or fibril?at* or arr?yth?m* or atrial or atrium or atria).mp. |
| 53 | ((recurr* or persistent* or long-dur* or long-stand* or longstand* or long-last* or longlast* or prolonged or continuing or chronic* or refractory or non-valv* or nonvalv* or nonparoxysm* or non-paroxysm*) adj3 AF).tw. |
| 54 | (LPAF or LSPAF or LSP-AF or PsAF or Ps-AF or R-AF or PerAF or Per-Af or CPAF).tw. |
| 55 | 23 or 24 or 25 or 26 or 27 or 28 or 29 or 30 or 31 or 32 or 33 or 34 or 35 or 36 or 37 or 38 or 39 or 40 or 41 or 42 or 43 or 44 or 45 or 46 or 47 or 48 or 49 or 50 or 51 or 52 or 53 or 54 |
| 56 | 8 and 22 and 55 |
| 57 | limit 56 to human |

**Econlit,via ProQuest ®**

| **No.** | **Search** |
| --- | --- |
| 1 | (pharmacogenetic* OR pharmacogenomic* OR Precision Medicin* OR Individuali?ed Medicin* OR Personali?ed Medicin* OR Individuali?ed treatment* OR Personali?ed treatment* OR Personali?ed therap* OR Individuali?ed therap* OR genetic* screen* OR genetic test* OR genotype* OR genetic marker* OR genomic marker* OR gene* OR mutation* OR genetic analys* OR phenotype* OR genetic variation OR genomics) |
| 2 | (cardiovascular disease* OR coronary arter* disease OR Coronary Arteriosclero* OR Angina Pectoris OR Myocardial Infarct* OR Myocardial repercussion OR Myocardium OR Heart Attack* OR Stroke OR Heart Failure* OR hypertensi* OR high blood pressure OR Rheumatic Heart Disease* OR Cardiomyopath* OR Heart Valve Disease* OR Myocarditis OR Aortic Aneurysm* OR Peripheral Arterial Disease* OR Peripheral Vascular Disease* OR Coronary Disease* OR Arteriosclero* OR Venous Thrombo* OR Acute rheumatic fever OR pericarditis OR endocarditis OR rheumatic heart disease* OR heart disease* OR isch?emic heart disease* OR Myocardial Ischemia OR cerebrovascular disease* OR Cerebrovascular Disorder* OR phlebitis OR thrombophlebitis OR hypotension OR Rheumatic chorea OR chorea OR Coronary thrombo* OR embolism* OR Atheroscleros* OR Mitral steno* OR Mitral Valve Steno* OR atrial fib* OR Atrial fibrillation OR atrial flutter OR atrial tachycardia OR paroxysmal atrial OR supraventricular tachycardia* OR Arrhythmia) |
| 3 | 1 AND 2 |

**Web of Science Core Collection**

| **No.** | **Search** |
| --- | --- |
| # 1 | TS=("cost benefit analys*" or CBA or "cost-benefit analysis") |
| # 2 | TS=(cost* NEAR/2 analys* or "costs and cost analysis") |
| # 3 | TS=(cost NEAR/2 effectiveness or "cost-effectiveness" or CEA) |
| # 4 | TS = ("cost-utility analys*" or "cost utility analys*" or CUA |
| # 5 | TS=(pharmacoeconomic* or "pharmaceutical economics") |
| # 6 | TS = ("econ* evaluat*") |
| # 7 | #6 OR #5 OR #4 OR #3 OR #2 OR #1 |
| # 8 | TS = (pharmacogenetic*) |
| # 9 | TS = (pharmacogenomic*) |
| # 10 | TS = ("precision medicin*") |
| # 11 | TS = ("individuali?ed medicin*") |
| # 12 | TS = ("individuali?ed treatment*") |
| # 13 | TS = ("personali?ed medicin*") |
| # 14 | TS = ("personali?ed treatment*") |
| # 15 | TS = ("personali?ed therap*") |
| # 16 | TS = ("genetic* screen*" "genetic* test*" or "genetic testing") |
| # 17 | TS = genotype* |
| # 18 | TS=(genomic*) |
| # 19 | TS=(genetic*) |
| # 20 | #19 OR #18 OR #17 OR #16 OR #15 OR #14 OR #13 OR #12 OR #11 OR #10 OR #9 OR #8 |
| # 21 | TS = ("cardiovascular disease*") |
| # 22 | TS = ("coronary arter* disease*") |
| # 23 | TS = ("coronary arteriosclero*") |
| # 24 | TS = ("angina pectoris") |
| # 25 | TS = ("myocardial infarct*") |
| # 26 | TS = ("myocardial reperfusion") |
| # 27 | TS = (myocardium) |
| # 28 | TS = ("heart attack*") |
| # 29 | TS = (stroke) |
| # 30 | TS = ("heart failure*") |
| # 31 | TS = (hypertensi* or hypertension) |
| # 32 | TS = ("high blood pressure") |
| # 33 | TS = ("rheumatic heart disease") |
| # 34 | TS = (cardiomyopath* or "diabetic cardiomyopathy") |
| # 35 | TS = ("heart valve disease*") |
| # 36 | TS = (myocarditis) |
| # 37 | TS = ("aortic aneurysm*") |
| # 38 | TS=("peripheral arterial disease*" or "peripheral vascular disease*" or "peripheral arterial disease" or "coronary disease*" or arteriosclerosis) |
| # 39 | TS = ("venous thrombosis" or "venous thrombo*") |
| # 40 | TS = ("acute rheumatic fever" or "rheumatic fever") |
| # 41 | TS = (pericarditis) |
| # 42 | TS = (endocarditis) |
| # 43 | TS = ("heart disease*" or "chronic rheumatic heart disease*") |
| # 44 | TS = ("isch?emic heart disease*" or "myocardial ischemia") |
| # 45 | TS = ("cerebrovascular disease*" or "cerebrovascular disorder*") |
| # 46 | TS = (phlebitis) |
| # 47 | TS = (thrombophlebitis) |
| # 48 | TS = (hypotension) |
| # 49 | TS = ("rheumatic chorea" or chorea) |
| # 50 | TS = ("coronary thrombo*" or "coronary thrombosis") |
| # 51 | TS = (embolism* or "pulmonary embolism*") |
| # 52 | TS = (atheroscleros?s) |
| # 53 | TS = ("mitral steno*" or "mitral valve stenosis") |
| # 54 | TS=(Atrial Fibrillation) |
| # 55 | TS=  (("atria*" or "atrium" or "auricular")  NEAR/6  fibril*) |
| # 56 | TS = (AF and  (flutter or "fibril?at*" or "arr?yth?m*" or atrial or atrium or atria) ) |
| # 57 | TS=  (("recurr*" or "persistent*" or "long-dur*" or "long-stand" or "longstand*" or "long-last*" or "longlast*" or prolonged or continuing or "chronic*" or refractory or "non-valv*" or "nonvalv*" or "nonparoxysm*" or "non-paroxysm*")  NEAR/3  AF) |
| # 58 | TS=(LPAF or  LSPAF  or  LSP-AF  or  PsAF  or  Ps-AF  or  R-AF  or  PerAF  or  Per-Af  or  CPAF) |
| # 59 | #58 OR #57 OR #56 OR #55 OR #54 OR #53 OR #52 OR #51 OR #50 OR #49 OR #48 OR #47 OR #46 OR #45 OR #44 OR #43 OR #42 OR #41 OR #40 OR #39 OR #38 OR #37 OR #36 OR #35 OR #34 OR #33 OR #32 OR #31 OR #30 OR #29 OR #28 OR #27 OR #26 OR #25 OR #24 OR #23 OR #22 OR #21 |
| # 60 | #59 AND #20 AND #7 |

**NHSEED database and HTA database via University of York Centre for Reviews & Dissemination** [**https://www.crd.york.ac.uk/CRDWeb/**](https://www.crd.york.ac.uk/CRDWeb/)

| **No.** | **Search** |
| --- | --- |
| 1 | MeSH DESCRIPTOR Pharmacogenetics EXPLODE ALL TREES IN NHSEED,HTA |
| 2 | MeSH DESCRIPTOR Pharmacogenomic Testing EXPLODE ALL TREES IN NHSEED,HTA |
| 3 | ((((Precision Medicin*)) and ((Economic evaluation:ZDT and Bibliographic:ZPS) OR (Economic evaluation:ZDT and Abstract:ZPS) OR Project record:ZDT OR Full publication record:ZDT) )) and ((Economic evaluation:ZDT and Bibliographic:ZPS) OR (Economic evaluation:ZDT and Abstract:ZPS) OR Project record:ZDT OR Full publication record:ZDT) IN NHSEED, HTA |
| 4 | MeSH DESCRIPTOR Precision Medicine EXPLODE ALL TREES IN NHSEED,HTA |
| 5 | ((((Individuali?ed Medicin*)) and ((Economic evaluation:ZDT and Bibliographic:ZPS) OR (Economic evaluation:ZDT and Abstract:ZPS) OR Project record:ZDT OR Full publication record:ZDT))) and ((Economic evaluation:ZDT and Bibliographic:ZPS) OR (Economic evaluation:ZDT and Abstract:ZPS) OR Project record:ZDT OR Full publication record:ZDT) IN NHSEED, HTA |
| 6 | ((((Individuali?ed treatment*)) and ((Economic evaluation:ZDT and Bibliographic:ZPS) OR (Economic evaluation:ZDT and Abstract:ZPS) OR Project record:ZDT OR Full publication record:ZDT) )) and ((Economic evaluation:ZDT and Bibliographic:ZPS) OR (Economic evaluation:ZDT and Abstract:ZPS) OR Project record:ZDT OR Full publication record:ZDT) IN NHSEED, HTA |
| 7 | ((((Individuali?ed therap*)) and ((Economic evaluation:ZDT and Bibliographic:ZPS) OR (Economic evaluation:ZDT and Abstract:ZPS) OR Project record:ZDT OR Full publication record:ZDT))) and ((Economic evaluation:ZDT and Bibliographic:ZPS) OR (Economic evaluation:ZDT and Abstract:ZPS) OR Project record:ZDT OR Full publication record:ZDT) IN NHSEED, HTA |
| 8 | ((((Personali?ed Medicin*)) and ((Economic evaluation:ZDT and Bibliographic:ZPS) OR (Economic evaluation:ZDT and Abstract:ZPS) OR Project record:ZDT OR Full publication record:ZDT) )) and ((Economic evaluation:ZDT and Bibliographic:ZPS) OR (Economic evaluation:ZDT and Abstract:ZPS) OR Project record:ZDT OR Full publication record:ZDT) IN NHSEED, HTA |
| 9 | ((((Personali?ed treatment*)) and ((Economic evaluation:ZDT and Bibliographic:ZPS) OR (Economic evaluation:ZDT and Abstract:ZPS) OR Project record:ZDT OR Full publication record:ZDT))) and ((Economic evaluation:ZDT and Bibliographic:ZPS) OR (Economic evaluation:ZDT and Abstract:ZPS) OR Project record:ZDT OR Full publication record:ZDT) IN NHSEED, HTA |
| 10 | ((((Personali?ed therap*)) and ((Economic evaluation:ZDT and Bibliographic:ZPS) OR (Economic evaluation:ZDT and Abstract:ZPS) OR Project record:ZDT OR Full publication record:ZDT) )) and ((Economic evaluation:ZDT and Bibliographic:ZPS) OR (Economic evaluation:ZDT and Abstract:ZPS) OR Project record:ZDT OR Full publication record:ZDT) IN NHSEED, HTA |
| 11 | ((((Genetic* screen*)) and ((Economic evaluation:ZDT and Bibliographic:ZPS) OR (Economic evaluation:ZDT and Abstract:ZPS) OR Project record:ZDT OR Full publication record:ZDT) )) and ((Economic evaluation:ZDT and Bibliographic:ZPS) OR (Economic evaluation:ZDT and Abstract:ZPS) OR Project record:ZDT OR Full publication record:ZDT) IN NHSEED, HTA |
| 12 | MeSH DESCRIPTOR Genetic Testing EXPLODE ALL TREES IN NHSEED,HTA |
| 13 | ((((Genetic* test*)) and ((Economic evaluation:ZDT and Bibliographic:ZPS) OR (Economic evaluation:ZDT and Abstract:ZPS) OR Project record:ZDT OR Full publication record:ZDT))) and ((Economic evaluation:ZDT and Bibliographic:ZPS) OR (Economic evaluation:ZDT and Abstract:ZPS) OR Project record:ZDT OR Full publication record:ZDT) IN NHSEED, HTA |
| 14 | ((((Genotype*)) and ((Economic evaluation:ZDT and Bibliographic:ZPS) OR (Economic evaluation:ZDT and Abstract:ZPS) OR Project record:ZDT OR Full publication record:ZDT))) and ((Economic evaluation:ZDT and Bibliographic:ZPS) OR (Economic evaluation:ZDT and Abstract:ZPS) OR Project record:ZDT OR Full publication record:ZDT) IN NHSEED, HTA |
| 15 | MeSH DESCRIPTOR Genotype EXPLODE ALL TREES IN NHSEED,HTA |
| 16 | MeSH DESCRIPTOR Genetic Markers EXPLODE ALL TREES IN NHSEED,HTA |
| 17 | MeSH DESCRIPTOR Genes EXPLODE ALL TREES IN NHSEED,HTA |
| 18 | ((((Mutation*)) and ((Economic evaluation:ZDT and Bibliographic:ZPS) OR (Economic evaluation:ZDT and Abstract:ZPS) OR Project record:ZDT OR Full publication record:ZDT))) and ((Economic evaluation:ZDT and Bibliographic:ZPS) OR (Economic evaluation:ZDT and Abstract:ZPS) OR Project record:ZDT OR Full publication record:ZDT) IN NHSEED, HTA |
| 19 | MeSH DESCRIPTOR Mutation EXPLODE ALL TREES IN NHSEED,HTA |
| 20 | MeSH DESCRIPTOR Cytogenetic Analysis EXPLODE ALL TREES IN NHSEED,HTA |
| 21 | ((((Phenotype*)) and ((Economic evaluation:ZDT and Bibliographic:ZPS) OR (Economic evaluation:ZDT and Abstract:ZPS) OR Project record:ZDT OR Full publication record:ZDT))) and ((Economic evaluation:ZDT and Bibliographic:ZPS) OR (Economic evaluation:ZDT and Abstract:ZPS) OR Project record:ZDT OR Full publication record:ZDT) IN NHSEED, HTA |
| 22 | MeSH DESCRIPTOR Phenotype EXPLODE ALL TREES IN NHSEED,HTA |
| 23 | MeSH DESCRIPTOR Genetic Variation EXPLODE ALL TREES IN NHSEED,HTA |
| 24 | MeSH DESCRIPTOR Genomics EXPLODE ALL TREES IN NHSEED,HTA |
| 25 | ((((Genet*)) and ((Economic evaluation:ZDT and Bibliographic:ZPS) OR (Economic evaluation:ZDT and Abstract:ZPS) OR Project record:ZDT OR Full publication record:ZDT))) and ((Economic evaluation:ZDT and Bibliographic:ZPS) OR (Economic evaluation:ZDT and Abstract:ZPS) OR Project record:ZDT OR Full publication record:ZDT) IN NHSEED, HTA |
| 26 | ((((Pharmacogen*)) and ((Economic evaluation:ZDT and Bibliographic:ZPS) OR (Economic evaluation:ZDT and Abstract:ZPS) OR Project record:ZDT OR Full publication record:ZDT))) and ((Economic evaluation:ZDT and Bibliographic:ZPS) OR (Economic evaluation:ZDT and Abstract:ZPS) OR Project record:ZDT OR Full publication record:ZDT) IN NHSEED, HTA |
| 27 | ((((genetic* marker*)) and ((Economic evaluation:ZDT and Bibliographic:ZPS) OR (Economic evaluation:ZDT and Abstract:ZPS) OR Project record:ZDT OR Full publication record:ZDT))) and ((Economic evaluation:ZDT and Bibliographic:ZPS) OR (Economic evaluation:ZDT and Abstract:ZPS) OR Project record:ZDT OR Full publication record:ZDT) IN NHSEED, HTA |
| 28 | ((((genomic* marker*)) and ((Economic evaluation:ZDT and Bibliographic:ZPS) OR (Economic evaluation:ZDT and Abstract:ZPS) OR Project record:ZDT OR Full publication record:ZDT))) and ((Economic evaluation:ZDT and Bibliographic:ZPS) OR (Economic evaluation:ZDT and Abstract:ZPS) OR Project record:ZDT OR Full publication record:ZDT) IN NHSEED, HTA |
| 29 | ((((genetic* analys*)) and ((Economic evaluation:ZDT and Bibliographic:ZPS) OR (Economic evaluation:ZDT and Abstract:ZPS) OR Project record:ZDT OR Full publication record:ZDT))) and ((Economic evaluation:ZDT and Bibliographic:ZPS) OR (Economic evaluation:ZDT and Abstract:ZPS) OR Project record:ZDT OR Full publication record:ZDT) IN NHSEED, HTA |
| 30 | ((((genetic* variation*)) and ((Economic evaluation:ZDT and Bibliographic:ZPS) OR (Economic evaluation:ZDT and Abstract:ZPS) OR Project record:ZDT OR Full publication record:ZDT))) and ((Economic evaluation:ZDT and Bibliographic:ZPS) OR (Economic evaluation:ZDT and Abstract:ZPS) OR Project record:ZDT OR Full publication record:ZDT) IN NHSEED, HTA |
| 31 | ((((genomic*)) and ((Economic evaluation:ZDT and Bibliographic:ZPS) OR (Economic evaluation:ZDT and Abstract:ZPS) OR Project record:ZDT OR Full publication record:ZDT) )) and ((Economic evaluation:ZDT and Bibliographic:ZPS) OR (Economic evaluation:ZDT and Abstract:ZPS) OR Project record:ZDT OR Full publication record:ZDT) IN NHSEED, HTA |
| 32 | #1 OR #2 OR #3 OR #4 OR #5 OR #6 OR #7 OR #8 OR #9 OR #10 OR #11 OR #12 OR #13 OR #14 OR #15 OR #16 OR #17 OR #18 OR #19 OR #20 OR #21 OR #22 OR #23 OR #24 OR #25 OR #26 OR #27 OR #28 OR #29 OR #30 OR #31 |
| 33 | ((((cardiovascular disease*)) and ((Economic evaluation:ZDT and Bibliographic:ZPS) OR (Economic evaluation:ZDT and Abstract:ZPS) OR Project record:ZDT OR Full publication record:ZDT))) and ((Economic evaluation:ZDT and Bibliographic:ZPS) OR (Economic evaluation:ZDT and Abstract:ZPS) OR Project record:ZDT OR Full publication record:ZDT) IN NHSEED, HTA |
| 34 | MeSH DESCRIPTOR Cardiovascular Diseases EXPLODE ALL TREES IN NHSEED,HTA |
| 35 | ((((coronary arter* disease*)) and ((Economic evaluation:ZDT and Bibliographic:ZPS) OR (Economic evaluation:ZDT and Abstract:ZPS) OR Project record:ZDT OR Full publication record:ZDT))) and ((Economic evaluation:ZDT and Bibliographic:ZPS) OR (Economic evaluation:ZDT and Abstract:ZPS) OR Project record:ZDT OR Full publication record:ZDT) IN NHSEED, HTA |
| 36 | MeSH DESCRIPTOR Coronary Artery Disease EXPLODE ALL TREES IN NHSEED,HTA |
| 37 | ((((Coronary Arteriosclero*)) and ((Economic evaluation:ZDT and Bibliographic:ZPS) OR (Economic evaluation:ZDT and Abstract:ZPS) OR Project record:ZDT OR Full publication record:ZDT) )) and ((Economic evaluation:ZDT and Bibliographic:ZPS) OR (Economic evaluation:ZDT and Abstract:ZPS) OR Project record:ZDT OR Full publication record:ZDT) IN NHSEED, HTA |
| 38 | ((((Angina Pectoris)) and ((Economic evaluation:ZDT and Bibliographic:ZPS) OR (Economic evaluation:ZDT and Abstract:ZPS) OR Project record:ZDT OR Full publication record:ZDT))) and ((Economic evaluation:ZDT and Bibliographic:ZPS) OR (Economic evaluation:ZDT and Abstract:ZPS) OR Project record:ZDT OR Full publication record:ZDT) IN NHSEED, HTA |
| 39 | MeSH DESCRIPTOR Angina Pectoris EXPLODE ALL TREES IN NHSEED,HTA |
| 40 | ((((Myocardial Infarct*)) and ((Economic evaluation:ZDT and Bibliographic:ZPS) OR (Economic evaluation:ZDT and Abstract:ZPS) OR Project record:ZDT OR Full publication record:ZDT))) and ((Economic evaluation:ZDT and Bibliographic:ZPS) OR (Economic evaluation:ZDT and Abstract:ZPS) OR Project record:ZDT OR Full publication record:ZDT) IN NHSEED, HTA |
| 41 | MeSH DESCRIPTOR Myocardial Infarction EXPLODE ALL TREES IN NHSEED,HTA |
| 42 | MeSH DESCRIPTOR Myocardial Reperfusion EXPLODE ALL TREES IN NHSEED,HTA |
| 43 | ((((Myocardium)) and ((Economic evaluation:ZDT and Bibliographic:ZPS) OR (Economic evaluation:ZDT and Abstract:ZPS) OR Project record:ZDT OR Full publication record:ZDT) )) and ((Economic evaluation:ZDT and Bibliographic:ZPS) OR (Economic evaluation:ZDT and Abstract:ZPS) OR Project record:ZDT OR Full publication record:ZDT) IN NHSEED, HTA |
| 44 | MeSH DESCRIPTOR Myocardium EXPLODE ALL TREES IN NHSEED,HTA |
| 45 | ((((Heart Attack*)) and ((Economic evaluation:ZDT and Bibliographic:ZPS) OR (Economic evaluation:ZDT and Abstract:ZPS) OR Project record:ZDT OR Full publication record:ZDT) )) and ((Economic evaluation:ZDT and Bibliographic:ZPS) OR (Economic evaluation:ZDT and Abstract:ZPS) OR Project record:ZDT OR Full publication record:ZDT) IN NHSEED, HTA |
| 46 | MeSH DESCRIPTOR Stroke EXPLODE ALL TREES IN NHSEED,HTA |
| 47 | ((((Heart Failure*)) and ((Economic evaluation:ZDT and Bibliographic:ZPS) OR (Economic evaluation:ZDT and Abstract:ZPS) OR Project record:ZDT OR Full publication record:ZDT))) and ((Economic evaluation:ZDT and Bibliographic:ZPS) OR (Economic evaluation:ZDT and Abstract:ZPS) OR Project record:ZDT OR Full publication record:ZDT) IN NHSEED, HTA |
| 48 | MeSH DESCRIPTOR Heart Failure EXPLODE ALL TREES IN NHSEED,HTA |
| 49 | ((((Hypertensi*)) and ((Economic evaluation:ZDT and Bibliographic:ZPS) OR (Economic evaluation:ZDT and Abstract:ZPS) OR Project record:ZDT OR Full publication record:ZDT))) and ((Economic evaluation:ZDT and Bibliographic:ZPS) OR (Economic evaluation:ZDT and Abstract:ZPS) OR Project record:ZDT OR Full publication record:ZDT) IN NHSEED, HTA |
| 50 | MeSH DESCRIPTOR Familial Primary Pulmonary Hypertension EXPLODE ALL TREES IN NHSEED,HTA |
| 51 | MeSH DESCRIPTOR Hypertension EXPLODE ALL TREES IN NHSEED,HTA |
| 52 | MeSH DESCRIPTOR Hypertension, Malignant EXPLODE ALL TREES IN NHSEED,HTA |
| 53 | MeSH DESCRIPTOR Hypertension, Portal EXPLODE ALL TREES IN NHSEED,HTA |
| 54 | MeSH DESCRIPTOR Hypertension, Pregnancy-Induced EXPLODE ALL TREES IN NHSEED,HTA |
| 55 | MeSH DESCRIPTOR Hypertension, Pulmonary EXPLODE ALL TREES IN NHSEED,HTA |
| 56 | MeSH DESCRIPTOR Hypertension, Renal EXPLODE ALL TREES IN NHSEED,HTA |
| 57 | MeSH DESCRIPTOR Hypertension, Renovascular EXPLODE ALL TREES IN NHSEED,HTA |
| 58 | MeSH DESCRIPTOR Intracranial Hypertension EXPLODE ALL TREES IN NHSEED,HTA |
| 59 | MeSH DESCRIPTOR Masked Hypertension EXPLODE ALL TREES IN NHSEED,HTA |
| 60 | MeSH DESCRIPTOR White Coat Hypertension EXPLODE ALL TREES IN NHSEED,HTA |
| 61 | ((((high blood pressure)) and ((Economic evaluation:ZDT and Bibliographic:ZPS) OR (Economic evaluation:ZDT and Abstract:ZPS) OR Project record:ZDT OR Full publication record:ZDT) )) and ((Economic evaluation:ZDT and Bibliographic:ZPS) OR (Economic evaluation:ZDT and Abstract:ZPS) OR Project record:ZDT OR Full publication record:ZDT) IN NHSEED, HTA |
| 62 | ((((Rheumatic Heart Disease*)) and ((Economic evaluation:ZDT and Bibliographic:ZPS) OR (Economic evaluation:ZDT and Abstract:ZPS) OR Project record:ZDT OR Full publication record:ZDT))) and ((Economic evaluation:ZDT and Bibliographic:ZPS) OR (Economic evaluation:ZDT and Abstract:ZPS) OR Project record:ZDT OR Full publication record:ZDT) IN NHSEED, HTA |
| 63 | MeSH DESCRIPTOR Rheumatic Heart Disease EXPLODE ALL TREES IN NHSEED,HTA |
| 64 | ((((Cardiomyopath*)) and ((Economic evaluation:ZDT and Bibliographic:ZPS) OR (Economic evaluation:ZDT and Abstract:ZPS) OR Project record:ZDT OR Full publication record:ZDT))) and ((Economic evaluation:ZDT and Bibliographic:ZPS) OR (Economic evaluation:ZDT and Abstract:ZPS) OR Project record:ZDT OR Full publication record:ZDT) IN NHSEED, HTA |
| 65 | MeSH DESCRIPTOR Cardiomyopathies EXPLODE ALL TREES IN NHSEED,HTA |
| 66 | MeSH DESCRIPTOR Cardiomyopathy, Dilated EXPLODE ALL TREES IN NHSEED,HTA |
| 67 | MeSH DESCRIPTOR Cardiomyopathy, Hypertrophic EXPLODE ALL TREES IN NHSEED,HTA |
| 68 | MeSH DESCRIPTOR Cardiomyopathy, Hypertrophic, Familial EXPLODE ALL TREES IN NHSEED,HTA |
| 69 | MeSH DESCRIPTOR Cardiomyopathy, Restrictive EXPLODE ALL TREES IN NHSEED,HTA |
| 70 | MeSH DESCRIPTOR Diabetic Cardiomyopathies EXPLODE ALL TREES IN NHSEED,HTA |
| 71 | ((((Heart Valve Disease*)) and ((Economic evaluation:ZDT and Bibliographic:ZPS) OR (Economic evaluation:ZDT and Abstract:ZPS) OR Project record:ZDT OR Full publication record:ZDT) )) and ((Economic evaluation:ZDT and Bibliographic:ZPS) OR (Economic evaluation:ZDT and Abstract:ZPS) OR Project record:ZDT OR Full publication record:ZDT) IN NHSEED, HTA |
| 72 | MeSH DESCRIPTOR Heart Valve Diseases EXPLODE ALL TREES IN NHSEED,HTA |
| 73 | ((((Myocarditis)) and ((Economic evaluation:ZDT and Bibliographic:ZPS) OR (Economic evaluation:ZDT and Abstract:ZPS) OR Project record:ZDT OR Full publication record:ZDT))) and ((Economic evaluation:ZDT and Bibliographic:ZPS) OR (Economic evaluation:ZDT and Abstract:ZPS) OR Project record:ZDT OR Full publication record:ZDT) IN NHSEED, HTA |
| 74 | MeSH DESCRIPTOR Myocarditis EXPLODE ALL TREES IN NHSEED,HTA |
| 75 | ((((Aortic Aneurysm*)) and ((Economic evaluation:ZDT and Bibliographic:ZPS) OR (Economic evaluation:ZDT and Abstract:ZPS) OR Project record:ZDT OR Full publication record:ZDT))) and ((Economic evaluation:ZDT and Bibliographic:ZPS) OR (Economic evaluation:ZDT and Abstract:ZPS) OR Project record:ZDT OR Full publication record:ZDT) IN NHSEED, HTA |
| 76 | MeSH DESCRIPTOR Aortic Aneurysm EXPLODE ALL TREES IN NHSEED,HTA |
| 77 | ((((Peripheral Arterial Disease*)) and ((Economic evaluation:ZDT and Bibliographic:ZPS) OR (Economic evaluation:ZDT and Abstract:ZPS) OR Project record:ZDT OR Full publication record:ZDT))) and ((Economic evaluation:ZDT and Bibliographic:ZPS) OR (Economic evaluation:ZDT and Abstract:ZPS) OR Project record:ZDT OR Full publication record:ZDT) IN NHSEED, HTA |
| 78 | MeSH DESCRIPTOR Peripheral Arterial Disease EXPLODE ALL TREES IN NHSEED,HTA |
| 79 | ((((Peripheral Vascular Disease*)) and ((Economic evaluation:ZDT and Bibliographic:ZPS) OR (Economic evaluation:ZDT and Abstract:ZPS) OR Project record:ZDT OR Full publication record:ZDT))) and ((Economic evaluation:ZDT and Bibliographic:ZPS) OR (Economic evaluation:ZDT and Abstract:ZPS) OR Project record:ZDT OR Full publication record:ZDT) IN NHSEED, HTA |
| 80 | MeSH DESCRIPTOR Peripheral Vascular Diseases EXPLODE ALL TREES IN NHSEED,HTA |
| 81 | ((((Coronary Disease*)) and ((Economic evaluation:ZDT and Bibliographic:ZPS) OR (Economic evaluation:ZDT and Abstract:ZPS) OR Project record:ZDT OR Full publication record:ZDT))) and ((Economic evaluation:ZDT and Bibliographic:ZPS) OR (Economic evaluation:ZDT and Abstract:ZPS) OR Project record:ZDT OR Full publication record:ZDT) IN NHSEED, HTA |
| 82 | MeSH DESCRIPTOR Coronary Disease EXPLODE ALL TREES IN NHSEED,HTA |
| 83 | MeSH DESCRIPTOR Arteriosclerosis EXPLODE ALL TREES IN NHSEED,HTA |
| 84 | MeSH DESCRIPTOR Intracranial Arteriosclerosis EXPLODE ALL TREES IN NHSEED,HTA |
| 85 | ((((Arterioscleros?s)) and ((Economic evaluation:ZDT and Bibliographic:ZPS) OR (Economic evaluation:ZDT and Abstract:ZPS) OR Project record:ZDT OR Full publication record:ZDT))) and ((Economic evaluation:ZDT and Bibliographic:ZPS) OR (Economic evaluation:ZDT and Abstract:ZPS) OR Project record:ZDT OR Full publication record:ZDT) IN NHSEED, HTA |
| 86 | ((((Venous Thrombo*)) and ((Economic evaluation:ZDT and Bibliographic:ZPS) OR (Economic evaluation:ZDT and Abstract:ZPS) OR Project record:ZDT OR Full publication record:ZDT) )) and ((Economic evaluation:ZDT and Bibliographic:ZPS) OR (Economic evaluation:ZDT and Abstract:ZPS) OR Project record:ZDT OR Full publication record:ZDT) IN NHSEED, HTA |
| 87 | MeSH DESCRIPTOR Venous Thrombosis EXPLODE ALL TREES IN NHSEED,HTA |
| 88 | MeSH DESCRIPTOR Venous Thromboembolism EXPLODE ALL TREES IN NHSEED,HTA |
| 89 | ((((Acute rheumatic fever)) and ((Economic evaluation:ZDT and Bibliographic:ZPS) OR (Economic evaluation:ZDT and Abstract:ZPS) OR Project record:ZDT OR Full publication record:ZDT))) and ((Economic evaluation:ZDT and Bibliographic:ZPS) OR (Economic evaluation:ZDT and Abstract:ZPS) OR Project record:ZDT OR Full publication record:ZDT) IN NHSEED, HTA |
| 90 | MeSH DESCRIPTOR Rheumatic Fever EXPLODE ALL TREES IN NHSEED,HTA |
| 91 | ((((pericarditis)) and ((Economic evaluation:ZDT and Bibliographic:ZPS) OR (Economic evaluation:ZDT and Abstract:ZPS) OR Project record:ZDT OR Full publication record:ZDT))) and ((Economic evaluation:ZDT and Bibliographic:ZPS) OR (Economic evaluation:ZDT and Abstract:ZPS) OR Project record:ZDT OR Full publication record:ZDT) IN NHSEED, HTA |
| 92 | MeSH DESCRIPTOR Pericarditis EXPLODE ALL TREES IN NHSEED,HTA |
| 93 | ((((endocarditis)) and ((Economic evaluation:ZDT and Bibliographic:ZPS) OR (Economic evaluation:ZDT and Abstract:ZPS) OR Project record:ZDT OR Full publication record:ZDT))) and ((Economic evaluation:ZDT and Bibliographic:ZPS) OR (Economic evaluation:ZDT and Abstract:ZPS) OR Project record:ZDT OR Full publication record:ZDT) IN NHSEED, HTA |
| 94 | MeSH DESCRIPTOR Endocarditis EXPLODE ALL TREES IN NHSEED,HTA |
| 95 | ((((Heart Disease*)) and ((Economic evaluation:ZDT and Bibliographic:ZPS) OR (Economic evaluation:ZDT and Abstract:ZPS) OR Project record:ZDT OR Full publication record:ZDT))) and ((Economic evaluation:ZDT and Bibliographic:ZPS) OR (Economic evaluation:ZDT and Abstract:ZPS) OR Project record:ZDT OR Full publication record:ZDT) IN NHSEED, HTA |
| 96 | MeSH DESCRIPTOR Heart Diseases EXPLODE ALL TREES IN NHSEED,HTA |
| 97 | MeSH DESCRIPTOR Pulmonary Heart Disease EXPLODE ALL TREES IN NHSEED,HTA |
| 98 | ((((chronic rheumatic heart disease*)) and ((Economic evaluation:ZDT and Bibliographic:ZPS) OR (Economic evaluation:ZDT and Abstract:ZPS) OR Project record:ZDT OR Full publication record:ZDT))) and ((Economic evaluation:ZDT and Bibliographic:ZPS) OR (Economic evaluation:ZDT and Abstract:ZPS) OR Project record:ZDT OR Full publication record:ZDT) IN NHSEED, HTA |
| 99 | ((((isch?emic heart disease*)) and ((Economic evaluation:ZDT and Bibliographic:ZPS) OR (Economic evaluation:ZDT and Abstract:ZPS) OR Project record:ZDT OR Full publication record:ZDT) )) and ((Economic evaluation:ZDT and Bibliographic:ZPS) OR (Economic evaluation:ZDT and Abstract:ZPS) OR Project record:ZDT OR Full publication record:ZDT) IN NHSEED, HTA |
| 100 | MeSH DESCRIPTOR Myocardial Ischemia EXPLODE ALL TREES IN NHSEED,HTA |
| 101 | ((((cerebrovascular disease*)) and ((Economic evaluation:ZDT and Bibliographic:ZPS) OR (Economic evaluation:ZDT and Abstract:ZPS) OR Project record:ZDT OR Full publication record:ZDT))) and ((Economic evaluation:ZDT and Bibliographic:ZPS) OR (Economic evaluation:ZDT and Abstract:ZPS) OR Project record:ZDT OR Full publication record:ZDT) IN NHSEED, HTA |
| 102 | ((((cerebrovascular disorder*)) and ((Economic evaluation:ZDT and Bibliographic:ZPS) OR (Economic evaluation:ZDT and Abstract:ZPS) OR Project record:ZDT OR Full publication record:ZDT) )) and ((Economic evaluation:ZDT and Bibliographic:ZPS) OR (Economic evaluation:ZDT and Abstract:ZPS) OR Project record:ZDT OR Full publication record:ZDT) IN NHSEED, HTA |
| 103 | MeSH DESCRIPTOR Cerebrovascular Disorders EXPLODE ALL TREES IN NHSEED,HTA |
| 104 | ((((phlebitis)) and ((Economic evaluation:ZDT and Bibliographic:ZPS) OR (Economic evaluation:ZDT and Abstract:ZPS) OR Project record:ZDT OR Full publication record:ZDT))) and ((Economic evaluation:ZDT and Bibliographic:ZPS) OR (Economic evaluation:ZDT and Abstract:ZPS) OR Project record:ZDT OR Full publication record:ZDT) IN NHSEED, HTA |
| 105 | MeSH DESCRIPTOR Phlebitis EXPLODE ALL TREES IN NHSEED,HTA |
| 106 | MeSH DESCRIPTOR Thrombophlebitis EXPLODE ALL TREES IN NHSEED,HTA |
| 107 | ((((thrombophlebitis)) and ((Economic evaluation:ZDT and Bibliographic:ZPS) OR (Economic evaluation:ZDT and Abstract:ZPS) OR Project record:ZDT OR Full publication record:ZDT))) and ((Economic evaluation:ZDT and Bibliographic:ZPS) OR (Economic evaluation:ZDT and Abstract:ZPS) OR Project record:ZDT OR Full publication record:ZDT) IN NHSEED, HTA |
| 108 | ((((hypotension)) and ((Economic evaluation:ZDT and Bibliographic:ZPS) OR (Economic evaluation:ZDT and Abstract:ZPS) OR Project record:ZDT OR Full publication record:ZDT))) and ((Economic evaluation:ZDT and Bibliographic:ZPS) OR (Economic evaluation:ZDT and Abstract:ZPS) OR Project record:ZDT OR Full publication record:ZDT) IN NHSEED, HTA |
| 109 | MeSH DESCRIPTOR Hypotension EXPLODE ALL TREES IN NHSEED,HTA |
| 110 | MeSH DESCRIPTOR Hypotension, Controlled EXPLODE ALL TREES IN NHSEED,HTA |
| 111 | MeSH DESCRIPTOR Hypotension, Orthostatic EXPLODE ALL TREES IN NHSEED,HTA |
| 112 | MeSH DESCRIPTOR Intracranial Hypotension EXPLODE ALL TREES IN NHSEED,HTA |
| 113 | ((((Rheumatic chorea)) and ((Economic evaluation:ZDT and Bibliographic:ZPS) OR (Economic evaluation:ZDT and Abstract:ZPS) OR Project record:ZDT OR Full publication record:ZDT) )) and ((Economic evaluation:ZDT and Bibliographic:ZPS) OR (Economic evaluation:ZDT and Abstract:ZPS) OR Project record:ZDT OR Full publication record:ZDT) IN NHSEED, HTA |
| 114 | MeSH DESCRIPTOR Chorea EXPLODE ALL TREES IN NHSEED,HTA |
| 115 | ((((chorea)) and ((Economic evaluation:ZDT and Bibliographic:ZPS) OR (Economic evaluation:ZDT and Abstract:ZPS) OR Project record:ZDT OR Full publication record:ZDT) )) and ((Economic evaluation:ZDT and Bibliographic:ZPS) OR (Economic evaluation:ZDT and Abstract:ZPS) OR Project record:ZDT OR Full publication record:ZDT) IN NHSEED, HTA |
| 116 | ((((Coronary thrombo*)) and ((Economic evaluation:ZDT and Bibliographic:ZPS) OR (Economic evaluation:ZDT and Abstract:ZPS) OR Project record:ZDT OR Full publication record:ZDT))) and ((Economic evaluation:ZDT and Bibliographic:ZPS) OR (Economic evaluation:ZDT and Abstract:ZPS) OR Project record:ZDT OR Full publication record:ZDT) IN NHSEED, HTA |
| 117 | MeSH DESCRIPTOR Coronary Thrombosis EXPLODE ALL TREES IN NHSEED,HTA |
| 118 | ((((embolism*)) and ((Economic evaluation:ZDT and Bibliographic:ZPS) OR (Economic evaluation:ZDT and Abstract:ZPS) OR Project record:ZDT OR Full publication record:ZDT))) and ((Economic evaluation:ZDT and Bibliographic:ZPS) OR (Economic evaluation:ZDT and Abstract:ZPS) OR Project record:ZDT OR Full publication record:ZDT) IN NHSEED, HTA |
| 119 | MeSH DESCRIPTOR Embolism EXPLODE ALL TREES IN NHSEED,HTA |
| 120 | MeSH DESCRIPTOR Embolism and Thrombosis EXPLODE ALL TREES IN NHSEED,HTA |
| 121 | MeSH DESCRIPTOR Embolism, Air EXPLODE ALL TREES IN NHSEED,HTA |
| 122 | MeSH DESCRIPTOR Embolism, Cholesterol EXPLODE ALL TREES IN NHSEED,HTA |
| 123 | MeSH DESCRIPTOR Embolism, Fat EXPLODE ALL TREES IN NHSEED,HTA |
| 124 | MeSH DESCRIPTOR Embolism, Paradoxical EXPLODE ALL TREES IN NHSEED,HTA |
| 125 | MeSH DESCRIPTOR Intracranial Embolism EXPLODE ALL TREES IN NHSEED,HTA |
| 126 | MeSH DESCRIPTOR Intracranial Embolism and Thrombosis EXPLODE ALL TREES IN NHSEED,HTA |
| 127 | MeSH DESCRIPTOR Pulmonary Embolism EXPLODE ALL TREES IN NHSEED,HTA |
| 128 | MeSH DESCRIPTOR Thromboembolism EXPLODE ALL TREES IN NHSEED,HTA |
| 129 | ((((Atheroscleros*)) and ((Economic evaluation:ZDT and Bibliographic:ZPS) OR (Economic evaluation:ZDT and Abstract:ZPS) OR Project record:ZDT OR Full publication record:ZDT))) and ((Economic evaluation:ZDT and Bibliographic:ZPS) OR (Economic evaluation:ZDT and Abstract:ZPS) OR Project record:ZDT OR Full publication record:ZDT) IN NHSEED, HTA |
| 130 | MeSH DESCRIPTOR Atherosclerosis EXPLODE ALL TREES IN NHSEED,HTA |
| 131 | MeSH DESCRIPTOR Carotid Artery Diseases EXPLODE ALL TREES IN NHSEED,HTA |
| 132 | MeSH DESCRIPTOR Intracranial Arteriosclerosis EXPLODE ALL TREES IN NHSEED,HTA |
| 133 | ((((Mitral steno*)) and ((Economic evaluation:ZDT and Bibliographic:ZPS) OR (Economic evaluation:ZDT and Abstract:ZPS) OR Project record:ZDT OR Full publication record:ZDT))) and ((Economic evaluation:ZDT and Bibliographic:ZPS) OR (Economic evaluation:ZDT and Abstract:ZPS) OR Project record:ZDT OR Full publication record:ZDT) IN NHSEED, HTA |
| 134 | MeSH DESCRIPTOR Mitral Valve Stenosis EXPLODE ALL TREES IN NHSEED,HTA |
| 135 | ((((Myocardial Reperfusion*)) and ((Economic evaluation:ZDT and Bibliographic:ZPS) OR (Economic evaluation:ZDT and Abstract:ZPS) OR Project record:ZDT OR Full publication record:ZDT))) and ((Economic evaluation:ZDT and Bibliographic:ZPS) OR (Economic evaluation:ZDT and Abstract:ZPS) OR Project record:ZDT OR Full publication record:ZDT) IN NHSEED, HTA |
| 136 | ((((Stroke*)) and ((Economic evaluation:ZDT and Bibliographic:ZPS) OR (Economic evaluation:ZDT and Abstract:ZPS) OR Project record:ZDT OR Full publication record:ZDT))) and ((Economic evaluation:ZDT and Bibliographic:ZPS) OR (Economic evaluation:ZDT and Abstract:ZPS) OR Project record:ZDT OR Full publication record:ZDT) IN NHSEED, HTA |
| 137 | ((Atrial Fibrillation)) and ((Economic evaluation:ZDT and Bibliographic:ZPS) OR (Economic evaluation:ZDT and Abstract:ZPS) OR Project record:ZDT OR Full publication record:ZDT) IN NHSEED, HTA |
| 138 | MeSH DESCRIPTOR Atrial Fibrillation EXPLODE ALL TREES IN NHSEED,HTA |
| 139 | ((Atrial Flutter)) and ((Economic evaluation:ZDT and Bibliographic:ZPS) OR (Economic evaluation:ZDT and Abstract:ZPS) OR Project record:ZDT OR Full publication record:ZDT) IN NHSEED, HTA |
| 140 | MeSH DESCRIPTOR Atrial Flutter EXPLODE ALL TREES IN NHSEED,HTA |
| 141 | ((LPAF OR LSP-AF OR PsAF or Ps-AF OR R-AF OR PerAF OR Per-Af OR CPAF)) and ((Economic evaluation:ZDT and Bibliographic:ZPS) OR (Economic evaluation:ZDT and Abstract:ZPS) OR Project record:ZDT OR Full publication record:ZDT) IN NHSEED, HTA |
| 142 | MeSH DESCRIPTOR Arrhythmias, Cardiac EXPLODE ALL TREES IN NHSEED,HTA |
| 143 | MeSH DESCRIPTOR Tachycardia, Ectopic Atrial EXPLODE ALL TREES IN NHSEED,HTA |
| 144 | #33 OR #34 OR #35 OR #36 OR #37 OR #38 OR #39 OR #40 OR #41 OR #42 OR #43 OR #44 OR #45 OR #46 OR #47 OR #48 OR #49 OR #50 OR #51 OR #52 OR #53 OR #54 OR #55 OR #56 OR #57 OR #58 OR #59 OR #60 OR #61 OR #62 OR #63 OR #64 OR #65 OR #66 OR #67 OR #68 OR #69 OR #70 OR #71 OR #72 OR #73 OR #74 OR #75 OR #76 OR #77 OR #78 OR #79 OR #80 OR #81 OR #82 OR #83 OR #84 OR #85 OR #86 OR #87 OR #88 OR #89 OR #90 OR #91 OR #92 OR #93 OR #94 OR #95 OR #96 OR #97 OR #98 OR #99 OR #100 OR #101 OR #102 OR #103 OR #104 OR #105 OR #106 OR #107 OR #108 OR #109 OR #110 OR #111 OR #112 OR #113 OR #114 OR #115 OR #116 OR #117 OR #118 OR #119 OR #120 OR #121 OR #122 OR #123 OR #124 OR #125 OR #126 OR #127 OR #128 OR #129 OR #130 OR #131 OR #132 OR #133 OR #134 OR #135 OR #136 OR #137 OR #138 OR #139 OR #140 OR #141 OR #142 OR #143 |
| 145 | #32 AND #144 |

# Appendix 4 Operationalization of Template for Intervention Description and Replication (TIDieR) checklist in our data extraction for testing and non-testing comparators

| **Category** | **TIDieR Items** | **Operationalization in current study for genetic testing** | **Operationalization in current study for non-testing comparator** |
| --- | --- | --- | --- |
| Brief Name | Provide the name or a phrase that describes the intervention. | Name of the gene(s) being tested and whether any biomarker(s) are tested alongside the gene.  Names (generic or commercial) of the genetic test | Name of the non-testing comparator e.g. "usual care" or "standard practice"  Name of the anticoagulant |
| Why | Describe any rationale, theory, or goal of the elements essential to the intervention. | Purpose or rationale for the genetic test | Purpose or rationale for the non-testing comparator |
| What | Materials: Describe any physical or informational materials used in the intervention, including those provided to participants or used in intervention delivery or in training of intervention providers. Provide information on where the materials can be accessed (e.g. online appendix, URL). | Any details on the test kit or test reagent | Any details on physical or informational materials for non-testing comparator, for patients or the providers delivering the comparator. |
|  | Procedures: Describe each of the procedures, activities, and/or processes used in the intervention, including any enabling or support activities. | Any procedures enabling or supporting the test e.g. genetic counselling.  If >1 gene tested or if any biomarker(s) are tested alongside the gene, whether the testing is sequential. | Any procedures enabling or supporting the non-testing comparator |
| Who provided | For each category of intervention provider (e.g. psychologist, nursing assistant), describe their expertise, background and any specific training given. | The type of providers involved in providing the genetic testing (e.g. collecting test samples, performing the test, interpreting the test results) and genetic counselling (if relevant) | The type of providers involved in providing the non-testing comparator. |
| How | Describe the modes of delivery (e.g. face-to-face or by some other mechanism, such as internet or telephone) of the intervention and whether it was provided individually or in a group. | Modes of testing e.g. saliva, blood samples, etc. | Mode of delivery of the non-testing comparator |
| Where | Describe the type(s) of location(s) where the intervention occurred, including any necessary infrastructure or relevant features. | Setting in which testing is provided e.g. primary care, hospital, community. | Setting in which the non-testing comparator is provided e.g. primary care, hospital, community. |
| When and How much | Describe the number of times the intervention was delivered and over what period of time including the number of sessions, their schedule, and their duration, intensity or dose. | Length of time testing is available for individuals  Length of time treatment is provided following genetic test | Length of time non-testing comparator is provided. |
| Tailoring | If the intervention was planned to be personalised, titrated or adapted, then describe what, why, when, and how. | Whether different test is provided to different (types of) individuals | Whether different treatment is provided to different (types of) patients in the non-testing comparator |
| Modifications | If the intervention was modified during the course of the study, describe the changes (what, why, when, and how). | Whether / how follow-up testing or treatment change based on the findings of the genetic test.  Name of anticoagulant. | Whether / how treatment change during the course of the trial / the time horizon of the model. |
| How Well | Planned: If intervention adherence or fidelity was assessed, describe how and by whom, and if any strategies were used to maintain or improve fidelity, describe them.  Actual: If intervention adherence or fidelity was assessed, describe the extent to which the intervention was delivered as planned. | Did the study assess (trial-based) / consider (model-based) uptake rate of the genetic test? | Did the study assess (trial-based) / consider (model-based) uptake of the non-testing comparator? |

# Appendix 5 Operationalization of the Second Panel’s reporting checklist for the present study

| **No** | **Elements / Items** | **Guidelines to support judgement (with page numbers and quotations from Second Panel's publications where available)** |
| --- | --- | --- |
| **Introduction** | |  |
| 1 | Background of the problem | Page 346 Neumann et al 2016.  "Provides contextual background by briefly describing the research question, the significance of the problem under investigation" |
| **Study Design & Scope** | | |
| 2 | Objectives | A study should provide a clear statement of study objectives |
| 3 | Audience | A study should state the audience for whom the writing was targeted.  Page 78 Neumann et al (eds) 2016  "Primary audiences for a CEA may include health plans, government entities, the US Public Health Service, or state health departments, as well as individual healthcare providers. Often there are additional decision makers who can use the same or similar information. Such secondary audiences may be groups who are not decision makers, but have an interest in the study results, such as patient advocacy groups, the press, the research community in the public and private sectors, or the general public." |
| 4 | Type of analysis | A study should provide a clear statement of the type of economic evaluations being performed e.g. cost-effectiveness analysis, cost utility analysis, cost benefit analysis  Page 78 - 79 Neumann et al (eds) 2016  "Before undertaking an analysis, the analyst, in consultation with relevant decision makers, should determine the type of analysis or analyses that will best illuminate the subject of the study. Many different forms of information can contribute to a decision. These may include a set of cost- effectiveness and related studies...... They are return- on- investment analysis, cost- minimization analysis, cost- consequence analysis, and cost– benefit analysis. Establishing which type of economic analysis is most useful depends on the objectives and scope of the analysis and the intervention under consideration." |
| 5 | Target population(s) | Page 87 Neumann et al (eds) 2016  "The target population is the population for whom the intervention is intended. Depending on the intervention, this may consist of individuals of a given age and sex, individuals living in a particular region, those with a specific disease, those with a certain risk profile, or groups defined by combinations of these characteristics." |
| 6 | Description of interventions and comparators (including "no intervention", if applicable | A study should describe the interventions and comparators being evaluated.  Page 87 Neumann et al (eds) 2016 on description of interventions.  "The types of intervention characteristics that will be important depend on the analysis. Some aspects of the intervention that the analyst should consider are these: the specific technologies used, the type of personnel delivering the intervention, the site of delivery, whether the intervention is bundled with other services, the timing of the intervention, and the healthcare system and country in which the intervention is delivered. The target population, discussed immediately below, is another critical aspect of the intervention definition."  Page 89 Neumann et al (eds) 2016 on description of comparators.  "As a rule and at a minimum, Reference Case analyses from the healthcare sector and societal perspectives should compare the intervention to relevant alternatives and to the existing practice for addressing the health problem (the status quo), which may itself be variable between locations, healthcare settings, and clinicians" |
| 7 | Other intervention descriptors (e.g. care setting, model of delivery, intensity and timing of intervention) | Page 350 Neumann et al (eds) 2016  "Characteristics of the intervention(s) to be specified include the care setting (location and type of institution— e.g., hospitals, ambulatory clinics, or primary care practices), the mode of service delivery (equipment, personnel, and other aspects of the strategy used), and details related to timing. Similar care should be devoted to describing the comparator intervention(s)." |
| 8 | Boundaries of the analysis (defining the scope or comprehensiveness of the study) | The potential cost and health impacts of the intervention should be explicitly described, including potential spillovers beyond the target population. This should be followed by a description on the extent to wthich the study accounts for the cost and health impacts. The description can be guided by the use of impact inventory proposed by the Second Panel.  Page 93 Neumann et al (eds) 2016  "Defining the boundaries of a study can be thought of as drawing a circle around the study to contain its scope. In circumscribing the study, the analyst must attempt to balance the need to capture all significant costs and effects of the intervention that will be relevant to the decision maker with the need to contain the study to the form of a manageable and feasible project. Considering the elements of the Impact Inventory when defining the scope of the analysis can help significantly with these tasks by elucidating the healthcare and non- healthcare sector effects to be included and measured in the analysis, while also identifying effects up front that may be difficult to measure quantitatively but should be acknowledged in the analysis" |
| 9 | Time horizon | The study should report the time horizon of the analyses. |
| 10 | Analytic perspectives (e.g., Reference Case perspectives included [healthcare sector, societal]; other perspectives such as employer or payer) | The study should report the analytic perspective. This also applies to studies funded by payers, governments or health technology assessment agencies. |
| 11 | Whether this analysis meets the requirements of the Reference Case | Neumann et al (eds) 2016 has a long list of Reference Case recommendations from Pages 369 - 382.  However, for the purpose of operationalizing this item we refer to the four recommendations reported in Sanders et al 2016: - Recommendation 1: Reference Cases and Perspectives - Recommendation 2: Health Care Sector Reference Case - Recommendation 3: Societal Reference Case - Recommendation 4: Reporting the Reference Cases  A study should perform its analysis in line with the four recommendations above, mostly requiring an analysis to include both healthcare and societal perspectives and to examine impact of an intervention beyond the healthcare sector. |
| 12 | Analysis plan | Page 94 Neumann et al (eds) 2016.  "(1) The analyst, in consultation with subject experts and decision makers, must develop a conceptual model describing the intervention and its effects on health and non-health outcomes; (2) The analyst must determine how to collect the data on costs, health effects, non-health effects, and preferences for health effects for the intervention and the relevant comparators from the perspectives selected; (3) The analyst must develop the analytic methods to combine the information appropriately into a CEA." |
| 13 | Trial- based analysis or model- based analysis. | The study should state whether it conducts a trial-based economic evaluation or model-based economic evaluation |
| **Methods & Data** | | |
|  |  | If model-based, answer items 14-18 |
| 14 | Description of event pathway/ model (describe condition or disease and the health states included) | A study should describe the event pathways i.e. the health conditions or disease / health states that follows the intervention and the comparator arms.  Page 96 Neumann et al (eds) 2016   "While the event pathway is generally constructed to represent health effects, depicting health states and events that have an impact on health, it also reflects the cascade of cost implications resulting from an intervention. The same events that cause changes in the health state of an individual generally trigger costs. The screening intervention that uncovers disease, for example, requires a visit to a clinician or other screening site, expends the patient’s time, and uses healthcare resources including a clinician’s time and laboratory tests. When costs arise from an event that is not explicit in a “clinical” event pathway— such as when a person moves from an acute care facility to a rehabilitation hospital without a change in health status— it may be useful to represent the change as a separate step or “state” in the pathway. Decision trees or probability trees may be used to represent schematically the conceptual model." |
| 15 | Diagram of event pathway/ model (depicting the sequencing and possible transitions among the health states included) | Page 352 Neumann et al (eds) 2016  "A diagram illustrating the event pathway is recommended." |
| 16 | Description of model used (e.g., decision tree, state transition, microsimulation) | A study should describe the type of models used. |
| 17 | Modelling assumptions | A study should describe the assumptions used in using the data and / or in the model. |
| 18 | Software used | A study should state the software used for modelling |
| 19 | Identification of key outcomes | A study should describe the outcome measures used. |
| 20 | Complete information on sources of effectiveness data, cost data, and preference weights | A study should cite or state the sources of all input data used in the model |
| 21 | Methods for obtaining estimates of effectiveness including approach(es) used for evidence synthesis. | Page 353 Neumann et al (eds) 2016.  "The analyst may discuss randomization, sample size and representativeness, and other aspects of study design and interpretation, such as the magnitude of effect sizes and confidence intervals for specific study parameters. Analysts should include detail of the evidence synthesis in the technical appendix."  "If a systematic review was performed specifically for the CEA, then details on the search strategy, inclusion/ exclusion criteria, and data analysis methods will be needed (and typically are presented in the technical appendix)."  "If the CEA used findings from an existing systematic review, analysts should discuss the strength of the evidence, the overall quality of the review, and its applicability to the population/ interventions of interest." |
| 22 | Methods for obtaining estimates of costs and preference weights | Page 355 Neumann et al (eds) 2016  "The general approach used to quantify each type of cost should be identified (e.g., as micro- costing or gross costing). Reasonably detailed information on these types of costs should be provided in the journal article, preferably in tables. If the analyst has separately measured and valued resources using a micro- costing approach, the table should report both the cost per unit of each resource and the number of units consumed. The source of data for each estimate should be described, including the type of study, survey, or database from which data were derived; the characteristics of the source population, such as insurance status (e.g., if data are derived from administrative data); and geographic location. It is important to describe any adjustments made (e.g., use of ratios of cost to charge at a particular institution), and to detail any other methods or models used to estimate unit costs. The author should also specify adjustments for inflation and should state the year and currency in which costs are presented (e.g., 2015 US dollars). As for any category of data input, analysts should comment on the quality and appropriateness of data sources." |
| 23 | Critique of data quality | A study should comment or critique the quality of their data (or input data for model-based economic evaluations) and attempt to address uncertainty of low-quality data via sensitivity analyses.  Page 253 Neumann et al (eds) 2016 "Apart from a study- specific description, it is important to convey to users of the CEA the analysts’ judgments about the magnitude of biases in the included studies and in the evidence base as a whole, and about how transferable the results of individual studies would be to the context of the CEA model" |
| 24 | Statement of costing year; this is the year to which all costs have been adjusted for the analysis (e.g., 2016) | A study should state the costing year |
| 25 | Statement of method used to adjust costs for inflation | A study should state how they adjust costs for inflation e.g. the inflation rate |
| 26 | Statement of type of currency | A study should state currency they used and in the case of any currency conversion, the conversion rate used |
| 27 | Source and methods for obtaining expert judgment, if applicable | Page 355 Neumann et al (eds) 2016  "If a CEA relies on experts to provide parameter estimates for any category of model inputs, the analysts should describe the basis for selecting the content experts, the number and type of experts, and the process used to elicit their input. Researchers have published best practices for eliciting expert judgment (O’Hagan et al. 2006; Sullivan and Payne 2011), and analysts should describe the extent to which such methods were employed. Authors should also discuss the rationale for using experts (e.g., to reconcile conflicting data, to estimate parameters in an area with little or no available evidence) and provide an assessment of the quality of the approach. Given space limitations, this material may be included in the technical appendix." |
| 28 | Statement of discount rate(s) | A study should state the discount rates for both cost and effect, especially whether the discount rates differ for both cost and effect |
| **Impact Inventory** | | |
| 29 | Full accounting of consequences within and outside of the healthcare sector (refer below) | Whether the analysis accounts for all consequences within and outside of the healthcare sector |
| **Results** | |  |
| 30 | Results of model validation | Page 356 Neumann et al (eds) 2016  "Appropriate assessments will generally include presentation of intermediate modeling results. For example, in an analysis using QALYs, the author might also describe the model’s predictions of the number of episodes of influenza and hospitalized pneumonia occurring with and without an intervention. Appraisals of the model’s performance using varying assumptions will demonstrate that the model obtains predictable results. When a previously validated model is used, the authors may choose to cite previous articles that provide evidence of validity." |
| 31 | Reference Case results (discounted and undiscounted): total costs and effectiveness, incremental costs and effectiveness, incremental cost effectiveness, ratios, measure(s) of uncertainty | The study should present the results required by the four recommendations of the Second Panel.  This should include findings based on discounted and undiscounted cost and effects. In cases where discounting is not applicable (e.g. <1 year time horizon), then the other recommendations e.g. presentation of results for both healthcare and societal perspectives are still expected. |
| 32 | Disaggregated results for important categories of costs, outcomes, or both | Page 358 Neumann et al (eds) 2016  "The reporting of disaggregated results refers to the attribution of total costs or QALYs to intermediate categories associated with specific cost categories (e.g., intervention-specific or relating to the care of the condition, healthcare sector, or other sector) or intermediate health outcomes. A typical breakdown of costs would report intervention-specific and condition- related costs, along with more detailed categories as relevant (e.g., hospitalizations, outpatient visits). Other categories may be relevant depending on the decision context." |
| 33 | Results of sensitivity analysis | Page 360 Neumann et al (eds) 2016  "The journal article should include both deterministic and probabilistic sensitivity analyses when appropriate" |
| 34 | Other estimates of uncertainty | Other estimates of uncertainty not counted as deterministic sensitivity analyses, probabilistic sensitivity analyses and secondary analyses.  For example, value of information analyses. |
| 35 | Graphical representation of cost- effectiveness results | Page 360 Neumann et al (eds) 2016  "We recommend that ICERs be presented graphically with a plot of net costs and effectiveness. The resulting illustrations can be placed in the technical appendix if space limitations preclude their inclusion in the journal article." |
| 36 | Graphical representation of uncertainty analyses | Page 360 Neumann et al (eds) 2016  "We also recommend visual displays to enhance the user’s understanding of key sources of uncertainty. These displays can include tornado diagrams, plots of two- or three- way sensitivity analyses, scatter plots, cost- effectiveness acceptability curves, and other types of displays" |
| 37 | Aggregate cost and effectiveness information | Page 358 Neumann et al (eds) 2016  "The aggregate intervention costs implied by a CEA may inform (or constrain) adoption independent of the intervention’s cost- effectiveness. Similarly, the total magnitude of benefit (whether to society or an individual) can be a consideration in addition to an intervention’s cost- effectiveness. The aggregate cost is the present value of the expected program costs for a decision maker, such as a screening program for a state public health department. Start- up or fixed costs, which may not be included in the primary analysis, may be relevant to the decision context and can be reported as secondary outcomes." |
| 38 | Secondary analyses | Page 360 Neumann et al (eds) 2016  "Secondary analyses included in a journal article might include: Other perspectives, alternative discounting approaches, variations in study designs." |
| **Disclosures** | |  |
| 39 | Statement of any potential conflicts of interest relating to funding source, collaborations, or outside interests | A study should state any potential conflicts of interest |
| **Discussion** | |  |
| 40 | Summary of Reference Case results | A study should present a summary of reference case results in the discussion section. |
| 41 | Summary of sensitivity of results to assumptions and uncertainties in the analysis | A study should discuss whether / how the base case results were sensitive to the assumptions or uncertainties demonstrated via sensitivity analyses.  Page 361 Neumann et al (eds) 2016  "The results of the Reference Case analyses should be highlighted and summarized, including the sensitivity of results to key assumptions and estimates" |
| 42 | Discussion of the study results in the context of results of related CEAs | Page 361 Neumann et al (eds) 2016  "The Discussion section should include a brief review of CEA results of similar or related interventions." |
| 43 | Discussion of ethical implications (e.g., distributive implications relating to age, disability, or other characteristics of the population) | Page 361 Neumann et al (eds) 2016  "The reporting of ethical concerns in the journal article should relate to the decision context at hand….The journal article should identify any ethical issues salient for the context. The analyst should highlight areas where study design decisions, such as the exclusion of patient or caregiver time in the healthcare sector Reference Case, could have ethical implications." |
| 44 | Limitations of the study | Page 362 Neumann et al (eds) 2016  "The discussion of limitations should also highlight any specific issues with the data, evidence synthesis, modeling, or analysis. The discussion of limitations should highlight the analyst’s efforts to compensate for the study’s shortcomings." |
| 45 | Relevance of study results to specific policy questions or decisions | Page 361 Neumann et al (eds) 2016  "The Discussion section allows researchers to place the CEA results in the decision context(s) identified, including the relevance of the results for that decision. Given that many decision makers do not employ strict cost- effectiveness thresholds, the Discussion should explain how the Reference Case results and secondary analyses can support the decisions under consideration." |
|  | **Conclusion** |  |
| 46 | Conclusion | A study should state the conclusion.  Page 362 Neumann et al (eds) 2016  "The conclusions section should provide a succinct summary of the results from the decision maker’s context, along with clinical or policy findings. This section should also highlight future areas of research suggested by the analysis" |
|  | **Technical appendix** | |
| 47 | Technical appendix | Provision of technical appendix alongside the journal article.  Page 362 Neumann et al (eds) 2016  "The technical appendix should provide enough detail on methods and data sources so that, along with the main journal report, analysts can reasonably replicate the analysis." |

# Appendix 6 One-page summary of included studies

| First Author (Year) | Eckman et al 2002 |
| --- | --- |
| URL | <https://journals.sagepub.com/doi/abs/10.1177/0272989X0202200209> |
| Stated Study Objectives | To address the health effects and costs of secondary screening in patients who have already suffered from an episode of venous thromboembolism (VTE). |
| Sample Characteristics | At base case, the study simulates 35-year old US women who survived a 1st venous thromboembolic event before anticoagulation is started or while patients are still receiving warfarin.  In sensitivity analyses, the study examines how ICER would change according to age and sex. The study also examines whether testing would be cost-effective among Asian or African populations who have low prevalence of factor V Leiden. |
| Intervention | The study has two intervention arms.  Both arms involve testing for factor V Leiden, followed by 3-month (arm #1) and lifelong warfarin (arm #2). Only patients screened positive for resistance to activated protein C (the phenotype) will be offered the genetic test. |
| Comparator | The study has one comparator arm, which was no testing with 6-month warfarin (usual care). |
| Type of model | Markov cohort model on Decision Maker® software  1-year cycle length; no mention of half-cycle correction. |
| Time horizon | Lifetime |
| Perspective | Societal, according to the recommendation of 1^st^ Washington Panel of Cost Effectiveness in Health & Medicine 1996 |
| Currency (year) | USD 1999, discounted at 3% annually |
| Outcome measures | QALY gained, discounted at 3% annually |
| Willingness-to-pay threshold | USD 50,000 per QALY gained, no justification given on the choice of WTP threshold. |
| Main findings & conclusion | Testing followed by 3-year warfarin is cost-saving compared to no-testing at base case (assumed zero risk of recurrent VTE after 3 years).  Testing followed by 3-year warfarin is also cost saving at modified base case (assumed lower but not zero risk of recurrent VTE after 3 years) and is cost-effective at constant-risk case (same recurrent VTE risk throughout). |
| Findings of sensitivity analysis | The study reported deterministic sensitivity analyses for 4 variables that may change the main conclusion – prevalence of factor V Leiden, rate of recurrent VTE, efficacy of anticoagulation therapy and rate of major bleeding on warfarin. |
| Funding source | National Library of Medicine, Bethesda, MD. |
| Competing interest | No competing interest statement given. |
| Reporting quality | 26/47 (55.3%) “Yes”, 6/47 “Partial”, 15/47 “No” |
| Methodological quality | 14/20 (70.0%) “Yes / Rather Yes”, 5/20 “No / Rather No”, 1/20 “Unclear” |

| First Author (Year) | Rubio-Terres 2015 |
| --- | --- |
| URL | <https://www.ncbi.nlm.nih.gov/pmc/articles/PMC4376955/> |
| Stated Study Objectives | To conduct an economic analysis of risk assessment of VTE from the perspective of the Spanish National Health System with Thrombo inCode (a clinical–genetic function for assessing the risk of VTE) versus the conventional/standard method used to date (factor V Leiden and prothrombin G20210A) |
| Sample Characteristics | The study simulates 10,000 45-year old patients living in Spain (S. Pau) and France (Martha) suffering a first spontaneous VTE event. |
| Intervention | The study has one intervention arm.  In the intervention arm, patients are tested for thrombophilia using ThromboInCode ®, a proprietary test kit that is able to simultaneous detect alleles of 12 variants located in seven genes (factor V Leiden, prothrombin G20210A, FXII, FXIII, ABO, Serpin A10 and Serpin C1) associated with higher risk of VTE.  Those tested positive would be treated with 6-month warfarin at base case, and between 1 to 35 years in sensitivity analyses. |
| Comparator | The study has one comparator arm, which was testing for factor V Leiden and prothrombin G20210A only (usual care).  Those tested positive would be treated with 6-month warfarin at base case, and between 1 to 35 years in sensitivity analyses. |
| Type of model | Decision tree on DATA™ software for Windows, TreeAge |
| Time horizon | 35 years at base case, based on local life expectancy, with 1 year as sensitivity analysis |
| Perspective | Healthcare sector |
| Currency (year) | EUR 2013, discounted at 3.5% annually |
| Outcome measures | QALY gained; LY gained, discounted at 3.5% annually |
| Willingness-to-pay threshold | EUR 30,000 per QALY gained, according to a [Spanish review paper](http://scielo.isciii.es/scielo.php?script=sci_arttext&pid=S0213-91112002000400008) on efficiency of health technology in Spain |
| Main findings & conclusion | Testing using ThromboInCode was dominant regardless of the duration of simulation (1 to 35 years). |
| Findings of sensitivity analysis | Deterministic sensitivity analyses found that ThromboInCode was cost-effective or cost-saving for all variables tested.  Probabilistic analyses found that ThromboInCode was dominant in 100 % simulations in both S. Pau and Martha populations. |
| Funding source | No funding source declared |
| Competing interest | C. Rubio-Terre´s and D. Rubio-Rodrıguez received an honorarium from Ferrer inCode in connection with the development of this manuscript. J. Sala and A. Gracia were employees of Ferrer inCode at the time of preparing the manuscript. S. Pich and E. Salas were employees of Gendiag.exe at the time of preparing the manuscript. The remaining authors have no conflicts of interest. |
| Reporting quality | 28/47 (59.6%) “Yes”, 2/47 “Partial”, 17/47 “No” |
| Methodological quality | 17/20 (85.0%) “Yes / Rather Yes”, 3/20 “No / Rather No”, 0/20 “Unclear” |

| First Author (Year) | Marchetti et al 2001 |
| --- | --- |
| URL | <https://academic.oup.com/qjmed/article/94/7/365/1598582> |
| Stated Study Objectives | To compare the standard anticoagulant prophylaxis to the strategy of screening for factor V Leiden and prothrombin G20210A and extending anticoagulation only for double heterozygotes for these mutations. |
| Sample Characteristics | The study simulates 1000 60-year old men living in Italy with the first episode of DVT. |
| Intervention | The study has one interventional arm.  In the interventional arm, patients were tested for Factor V Leiden and Prothrombin G20210A. Those tested positive for double heterozygotes undergo 2-year warfarin. |
| Comparator | The study has one comparator arm, which was no-testing followed by 6-month warfarin. |
| Type of model | Markov cohort model on DATA™ software for Windows, TreeAge  6-month cycle length; no mention of half-cycle correction |
| Time horizon | Lifetime |
| Perspective | Societal |
| Currency (year) | USD 2000, discounted at 3% annually |
| Outcome measures | QALY gained; LY gained; VTE event averted; bleeding event averted, discounted at 3% annually |
| Willingness-to-pay threshold | USD 50,000 per QALY gained, based on Weinstein et al 1996 recommendations of the Panel on Cost‐Effectiveness in Health and Medicine |
| Main findings & conclusion | Testing of all patients with venous thromboembolism results in an ICER of USD 13,624 / QALY gained. |
| Findings of sensitivity analysis | The study reported three variables that may change the conclusion – prevalence of mutation, six-month rate of major bleeding and efficacy of warfarin prophylaxis. |
| Funding source | No funding source declared |
| Competing interest | No competing interest statement given. |
| Reporting quality | 19/46 (41.3%) “Yes”, 8/46 “Partial”, 19/46 “No”, excluding 1 “Not Applicable” |
| Methodological quality | 13/20 (65.0%) “Yes / Rather Yes”, 7/20 “No / Rather No”, 0/20 “Unclear” |

| First Author (Year) | Marchetti et al 2000 |
| --- | --- |
| URL | <https://www.thieme-connect.com/products/ejournals/pdf/10.1055/s-0037-1614110.pdf> |
| Stated Study Objectives | To evaluate the impact of screening for factor V Leiden mutation and standard management on both quality-adjusted life expectancy and lifelong costs among patients suffering a first episode of deep vein thrombosis (DVT). |
| Sample Characteristics | The study simulates 1000 60-year old men living in Italy suffering a first episode of deep vein thrombosis (DVT). |
| Intervention | The study has one interventional arm.  In the interventional arm, patients were tested for Factor V Leiden. Those tested positive undergo 2-year warfarin. |
| Comparator | The study has one comparator arm, which was no-testing followed by 6-month warfarin. |
| Type of model | Markov cohort model on DATA™ software for Windows, TreeAge  6-month cycle length; no mention of half-cycle correction |
| Time horizon | Lifetime |
| Perspective | Societal |
| Currency (year) | USD (cost year not stated), discounted at 3% annually |
| Outcome measures | QALY gained; LY gained; VTE event averted; bleeding event averted, discounted at 3% annually |
| Willingness-to-pay threshold | USD 50,000 per QALY gained, based on Weinstein et al 1996 recommendations of the Panel on Cost‐Effectiveness in Health and Medicine |
| Main findings & conclusion | Testing was a cost-effective strategy, with an ICER of USD 12,833 per QALY gained. |
| Findings of sensitivity analysis | The study reported six variables that may change the conclusion – rate of recurrent thromboembolism, rate of fatal bleeding on warfarin, efficacy of warfarin, compliance to warfarin, quality of life on warfarin. |
| Funding source | No funding source declared |
| Competing interest | No competing interest statement given. |
| Reporting quality | 22/46 (47.8%) “Yes”, 5/46 “Partial”, 19/46 “No”, excluding 1 “Not Applicable” |
| Methodological quality | 12/20 (60.0%) “Yes / Rather Yes”, 8/20 “No / Rather No”, 0/20 “Unclear” |

| First Author (Year) | You et al 2004 |
| --- | --- |
| URL | <https://www.thieme-connect.com/products/ejournals/abstract/10.1160/TH04-03-0161> |
| Stated Study Objectives | To evaluate the potential clinical and economic outcomes of using CYP2C9 genotype data to guide the management of anticoagulation therapy and to identify influential factors affecting the cost-effectiveness of this treatment scheme. |
| Sample Characteristics | The study simulates the costs and outcomes for 18-year old patients in the US newly started on warfarin therapy (100 patient-years). |
| Intervention | The study has one interventional arm.  In the interventional arm, patients were tested for CYP2C9 polymorphisms. Those tested positive would receive initial dose of warfarin based on demographic, clinical and pharmacogenetic (CYP2C9 genotypes). |
| Comparator | The study has one comparator arm, which was no-testing followed by normal initial dose of warfarin. |
| Type of model | Decision tree on DATA™ 3.5 software for Windows, TreeAge |
| Time horizon | 1 year |
| Perspective | Healthcare sector |
| Currency (year) | USD 2001, no discounting |
| Outcome measures | VTE event averted; bleeding event averted; total events averted, no discounting. |
| Willingness-to-pay threshold | No willingness-to-pay threshold |
| Main findings & conclusion | Testing for CYP2C9 polymorphisms followed by genotype-guided warfarin dosing results in 9.58 total events and incurs USD 155,700 whereas no-testing results in 10.48 total events and incurs USD 150,500, with USD 5,778 per additional major bleeding averted. The study did not conclude whether testing was cost-effective. |
| Findings of sensitivity analysis | The study reported that the model was sensitive to the variation of the cost and reduction of bleeding rate in the interventional arm. However, it did not conclude whether the variation crossed any willingness-to-pay threshold. |
| Funding source | School of Pharmacy, The Chinese University of Hong Kong |
| Competing interest | No competing interest statement given. |
| Reporting quality | 21/45 (46.7%) “Yes”, 4/45 “Partial”, 20/45 “No”, excluding 2 “Not Applicable” |
| Methodological quality | 11/20 (55.0%) “Yes / Rather Yes”, 8/20 “No / Rather No”, 1/20 “Unclear” |

| First Author (Year) | You et al 2009 |
| --- | --- |
| URL | <https://ascpt.onlinelibrary.wiley.com/doi/abs/10.1038/clpt.2009.104> |
| Stated Study Objectives | To evaluate the potential clinical and economic outcomes of the CYP2C9 and VKORC1 genotype-guided dosing algorithm in patients newly started on warfarin therapy and to identify factors affecting the cost-effectiveness of the dosing algorithm. |
| Sample Characteristics | The study simulates the costs and outcomes for 18-year old patients (number not stated) in the US in whom warfarin therapy is to be initiated. |
| Intervention | The study has one interventional arm.  In the interventional arm, patients were tested for CYP2C9 and VKORC1 genotypes. Those tested positive would receive initial dose of warfarin based on demographic, clinical, and pharmacogenetic (CYP2C9 and VKORC1 genotypes). |
| Comparator | The study has one comparator arm, which was no-testing followed by 10-mg warfarin dosing nomogram used in the Couma-Gen trial. |
| Type of model | Decision tree on TreeAge Pro 2008 and Microsoft Excel 2003 |
| Time horizon | 1 year |
| Perspective | Healthcare sector |
| Currency (year) | USD 2008, no discounting |
| Outcome measures | QALY gained; LY gained; total events averted, no discounting |
| Willingness-to-pay threshold | USD 50,000 per QALY gained, based on Weinstein et al 1996 recommendations of the Panel on Cost‐Effectiveness in Health and Medicine |
| Main findings & conclusion | Testing for CYP2C9 and VKORC1 genotypes followed by genotype-guided warfarin dosing was not cost-effective, with ICER > USD 50,000. |
| Findings of sensitivity analysis | The study reported that the main findings were sensitive to the cost of genetic testing, effectiveness of INR control of the genotype-guided dosing algorithm, percentage of high out-of-range INRs in standard dosing. |
| Funding source | Research Grants Council of the Hong Kong Special Administrative Region, China (project CUHK4519/06M) |
| Competing interest | The authors declared no conflict of interest. |
| Reporting quality | 27/46 (58.7%) “Yes”, 3/46 “Partial”, 16/46 “No”, excluding 1 “Not Applicable” |
| Methodological quality | 13/20 (65.0%) “Yes / Rather Yes”, 7/20 “No / Rather No”, 0/20 “Unclear” |

| First Author (Year) | Schalekamp et al 2006 |
| --- | --- |
| URL | <https://ascpt.onlinelibrary.wiley.com/doi/abs/10.1016/j.clpt.2006.03.008> |
| Stated Study Objectives | To evaluate the economic outcomes of CYP2C9 genotyping on the possibility to prevent major bleeding |
| Sample Characteristics | The study simulates patients in the Netherlands preceding or shortly after initiation of acenocoumarol (100 patient years). |
| Intervention | The study has one interventional arm.  In the interventional arm, patients were tested for CYP2C9*2 and CYP2C9*3 polymorphisms. Those tested positive would undergo more intensive monitoring of INR in the first year (25% more monitoring than comparator). |
| Comparator | The study has one comparator arm, which was no-testing followed by regular monitoring of INR (usual care). |
| Type of model | Decision tree on DATA™ 3.5 software for Windows, TreeAge |
| Time horizon | Not clearly stated |
| Perspective | Not clearly stated |
| Currency (year) | EUR 2004, no discounting |
| Outcome measures | Bleeding event averted, no discounting |
| Willingness-to-pay threshold | EUR 4,000 per major bleeding episode averted, based on the assumption that 20% to 24% of €20,000 per QALY gained recommended by Netherlands regulator would be acceptable. |
| Main findings & conclusion | In the base case where all patients were tested, the cost per major bleeding averted was EUR 4,233. In the alternative base case where patients with INR > 2.5 on Day 4 of anticoagulation were tested, the cost per major bleeding averted was EUR 2,210. |
| Findings of sensitivity analysis | The study reported six variables that may change conclusion – incidence rate of major bleeding episodes in wild type; relative risk reduction with known CYP2C9 genotype; relative risk of major bleeding in carriers of polymorphism compared to wild type patients; additional INR measurements; cost of major bleeding; and cost of genotyping. |
| Funding source | No funding source declared |
| Competing interest | None of the authors has a conflict of interest. |
| Reporting quality | 16/47 (34.0%) “Yes”, 7/47 “Partial”, 24/47 “No” |
| Methodological quality | 12/20 (60.0%) “Yes / Rather Yes”, 6/20 “No / Rather No”, 2/20 “Unclear” |

| First Author (Year) | O’Brien et al 2009 |
| --- | --- |
| URL | <https://www.sciencedirect.com/science/article/abs/pii/S0022347609000146> |
| Stated Study Objectives | To design a decision-analytic model addressing the cost-effectiveness of thrombophilia testing and treatment strategies in pediatric patients with a first episode of DVT. |
| Sample Characteristics | The study simulates paediatric patients 2 months – 18 years old (number not stated) in the US who survived their initial thrombotic event. |
| Intervention | The study has one interventional arm.  In the interventional arm, patients were tested for factor V Leiden, prothrombin G20210 mutation, and Protein C, Protein S, and antithrombin activity levels.  Those tested positive would undergo 6-month warfarin. |
| Comparator | The study has two comparator arms, which was no-testing followed by 3-month (arm #1) OR 6-month warfarin (arm #2). |
| Type of model | Markov cohort model on TreeAge Pro Suite 2006  1-month cycle length (inferred based on input data – monthly probability and monthly cost); no mention of half-cycle correction |
| Time horizon | 2 years |
| Perspective | Societal |
| Currency (year) | USD 2007, no discounting |
| Outcome measures | QALY gained, no discounting |
| Willingness-to-pay threshold | USD 50,000 per QALY gained, based on [Hirth et al 2000 review paper](https://journals.sagepub.com/doi/10.1177/0272989X0002000310) on willingness to pay for a quality-adjusted life year. |
| Main findings & conclusion | Universal thrombophilia testing after a first episode of thrombosis is not cost-effective when used solely to determine anticoagulation duration. |
| Findings of sensitivity analysis | The study reported that one-way sensitivity analyses of all model measures produced no scenarios that invalidated the key findings. |
| Funding source | American Society of Hematology Fellow Scholar Award |
| Competing interest | The authors declare no conflicts of interest. |
| Reporting quality | 24/46 (52.2%) “Yes”, 5/46 “Partial”, 17/46 “No”, excluding 1 “Not Applicable” |
| Methodological quality | 15/20 (75.0%) “Yes / Rather Yes”, 4/20 “No / Rather No”, 1/20 “Unclear” |

| First Author (Year) | Auerbach et al 2004 |
| --- | --- |
| URL | <https://www.amjmed.com/article/S0002-9343(04)00152-4/fulltext> |
| Stated Study Objectives | To determine which hypercoagulable conditions should be included in a test panel, which positive tests affected the choice of initial treatment for patients with idiopathic deep vein thrombosis, and also whether newer evidence regarding recurrence rates affected the clinical approach to testing and anticoagulation. |
| Sample Characteristics | The study simulates 40-year old patients (number not stated) in the US with idiopathic deep vein thrombosis (without a known inciting cause, such as immobilization, cancer, or pregnancy) |
| Intervention | The study has five international arms.  In all interventional arms, patients are tested for mutations in 3 genes - FVL, PT and dihydrofolate reductase, followed by anticardiolipin antibody, antithrombin III, and protein C and S levels after initial anticoagulation. Those tested positive then undergo 12-month (arm #1), 18-month (arm #2), 24-month (arm #3), 36-month (arm #4) or lifelong warfarin (arm #4). |
| Comparator | The study has five comparator arms, which has no testing with 6-month (usual care, arm #1), 12-month (arm #2), 18-month (arm #3), 24-month (arm #4) or 36-month (arm #5). |
| Type of model | Markov cohort model on TreeAge 3.5 for Windows  1-year cycle length; no mention of half-cycle correction |
| Time horizon | Lifetime |
| Perspective | Societal |
| Currency (year) | USD 2000, discounted at 3% annually |
| Outcome measures | QALY gained, discounted at 3% annually |
| Willingness-to-pay threshold | USD 50,000 per QALY gained, no justification given on the choice of WTP threshold. |
| Main findings & conclusion | Testing for hypercoagulable disorders in patients with idiopathic deep vein thrombosis followed by 2 years of anticoagulation in affected patients is cost-effective, compared to no-testing followed by 24-month warfarin. Other arms were either dominated or extendedly dominated. |
| Findings of sensitivity analysis | The study reported that varying probabilities, costs and QALY weights singly and in pairs (probability and costs of minor complications) did not produce a scenario that invalidated the base case findings. |
| Funding source | Dr Auerbach is supported by a Mentored Research Career Development Training Grant (HS11416) from the Agency for Healthcare Research and Quality, Rockville, Maryland. |
| Competing interest | No competing interest statement given. |
| Reporting quality | 24/46 (52.2%) “Yes”, 3/46 “Partial”, 19/46 “No”, excluding 1 “Not Applicable” |
| Methodological quality | 13/20 (65.0%) “Yes / Rather Yes”, 7/20 “No / Rather No”, 0/20 “Unclear” |

| First Author (Year) | Simpson et al 2009 |
| --- | --- |
| URL | <https://doi.org/10.3310/hta13020> |
| Stated Study Objectives | To assess whether thrombophilia testing following a venous thrombotic event is clinically effective and cost-effective in the management of thrombosis compared with no testing for thrombophilia. |
| Sample Characteristics | The study simulates 20,000 patients in the UK with idiopathic deep vein thrombosis or pulmonary embolism (confirmed by objective testing). Simulations were performed for 30-, 40-, 50-, 60- and 70-year olds men and women, with DVT or PE separately. |
| Intervention | The study has one interventional arm.  In the interventional arm, patients were tested for FVL, PTG20210A, AT deficiency, PC deficiency, PS deficiency, lupus anticoagulants and anticardiolipin antibodies, followed by 3-month, 10-year, 20-year or lifelong warfarin. |
| Comparator | The study has one comparator arm, which was no testing with 3-month warfarin (usual care). |
| Type of model | Discrete event simulation on Simul8© |
| Time horizon | Lifetime |
| Perspective | Healthcare sector |
| Currency (year) | GBP 2005-06, discounted at 3.5% annually |
| Outcome measures | QALY gained, discounted at 3.5% annually |
| Willingness-to-pay threshold | GBP 20,000 per QALY gained at base case, based on NICE recommendation, with GBP 30,000 per QALY gained at sensitivity analysis. |
| Main findings & conclusion | Testing on patients with PE has a mean cost per QALY of <GBP 20,000 regardless of sex or age. For men aged ≤69 years old with a previous DVT and for women aged ≤49 years old with a previous DVT, testing has a cost per QALY of <GBP 20,000. Thrombophilia testing is also indicated to be cost-effective in men aged >70 years with a previous DVT if a MAICER of GBP 30,000 per QALY is employed. |
| Findings of sensitivity analysis | In probabilistic analyses, testing was cost-effective in 30%-60% of simulation in different patient subgroups. The study did not perform any deterministic sensitivity analysis. |
| Funding source | Commissioned and funded by the HTA Programme on behalf of NICE as project number 06/66/01 |
| Competing interest | The authors declared no competing interests. |
| Reporting quality | 31/46 (67.4%) “Yes”, 2/46 “Partial”, 13/46 “No”, excluding 1 “Not Applicable”. |
| Methodological quality | 16/20 (80.0%) “Yes / Rather Yes”, 4/20 “No / Rather No”, 0/20 “Unclear” |

# Appendix 7 Impact of genetic-guided pharmacotherapy accounted for

| **No** | **Impact inventory** |  |  |  |  |  |  |  |  |  |  |  |  |
| --- | --- | --- | --- | --- | --- | --- | --- | --- | --- | --- | --- | --- | --- |
|  |  | | **Healthcare perspective** | | | | | **Societal perspective** | | | | |  |
|  | **Healthcare sector: Formal** | |  | | |  |  |  |  | | | |  |
| 1 | Health | Longevity effects |  |  |  | √ | √ |  |  |  | √ | √ |  |
| 2 |  | Health-related quality-of-life effects |  |  | √ | √ | √ | √ | √ | √ | √ | √ |  |
| 3 |  | Other health effects (e.g., adverse events and secondary transmissions of infections) | √ | √ |  | √ |  |  |  |  | √ | √ |  |
| 4 |  | Paid for by third- party payers |  |  |  |  |  |  |  |  |  |  |  |
| 5 |  | Paid for by patients out- of- pocket |  |  |  |  |  |  |  |  |  |  |  |
| 6 |  | Future related medical costs (payers and patients) |  |  | √ |  | √ | √ | √ |  | √ | √ |  |
| 7 |  | Future unrelated medical costs (payers and patients) |  |  |  |  |  |  |  |  |  |  |  |
|  | **Healthcare sector: Informal** | |  |  |  |  |  |  |  |  |  |  |  |
| 8 | Health | Patient time costs |  |  |  |  |  |  |  |  |  |  |  |
| 9 |  | Unpaid caregiver time costs |  |  |  |  |  |  |  |  |  |  |  |
| 10 |  | Transportation costs |  |  |  |  |  |  |  | √ |  |  |  |
|  | **Non-healthcare sector** | |  |  |  |  |  |  |  |  |  |  |  |
| 11 | Productivity | Labour market earnings lost |  |  |  |  |  |  |  | √ | √ | √ |  |
| 12 |  | Cost of unpaid lost productivity due to illness |  |  |  |  |  |  |  |  |  |  |  |
| 13 |  | Cost of uncompensated household production |  |  |  |  |  |  |  |  |  |  |  |
| 14 | Consumption | Future consumption unrelated to health |  |  |  |  |  |  |  |  |  |  |  |
| 15 | Social services | Cost of social services as part of intervention |  |  |  |  |  |  |  |  |  |  |  |
| 16 | Legal/criminal justice | Number of crimes related to intervention |  |  |  |  |  |  |  |  |  |  |  |
|  |  | Cost of crimes related to intervention |  |  |  |  |  |  |  |  |  |  |  |
| 18 | Education | Impact of intervention on educational achievement of population |  |  |  |  |  |  |  |  |  |  |  |
| 19 | Housing | Cost of intervention on home improvements (e.g., removing lead paint) |  |  |  |  |  |  |  |  |  |  |  |
| 20 | Environment | Production of toxic waste or pollution by intervention |  |  |  |  |  |  |  |  |  |  |  |
| 21 | Other (specify) | Other impacts |  |  |  |  |  |  |  |  |  |  |  |
|  |  |  |  |  |  |  |  |  |  |  |  |  |  |

*‘*√*’ indicates that the specific item in the impact inventory* ***is*** *accounted for in the study; ‘(blank)’ indicates that the specific item in the impact inventory* ***is not*** *accounted for in the study; ‘NA’ indicates that the specific item in the impact inventory is not applicable for the study.*

*a. This study did not specify a study perspective; we assumed healthcare perspective based on the input variables reported.*

# Appendix 8 Reporting quality ratings of included studies in full, based on the 2nd Washington Panel reporting checklist

|  | **Items (Are the following elements reported?)** | **[10]** | **[11]** | **[12]** | **[13]** | **[14]** | **[15]** | **[16]** | **[17]** | **[18]** | **[19]** | **Total “Yes”** |
| --- | --- | --- | --- | --- | --- | --- | --- | --- | --- | --- | --- | --- |
|  | **Introduction** |  |  |  |  |  |  |  |  |  |  |  |
| 1 | Background of the problem | Y | Y | P | Y | Y | Y | Y | Y | Y | Y | 9 |
|  |  |  |  |  |  |  |  |  |  |  |  |  |
|  | **Study design & scope** |  |  |  |  |  |  |  |  |  |  |  |
| 2 | Objectives | Y | Y | Y | Y | Y | Y | P | Y | Y | Y | 9 |
| 3 | Audience | N | N | N | N | N | N | N | N | N | Y | 0 |
| 4 | Type of analysis | Y | Y | Y | Y | Y | Y | Y | Y | Y | Y | 10 |
| 5 | Target population(s) | Y | Y | Y | Y | P | P | N | Y | Y | Y | 7 |
| 6 | Description of interventions and comparators (including "No intervention", if applicable) | Y | Y | Y | Y | Y | Y | Y | Y | Y | Y | 10 |
| 7 | Other intervention descriptors (e.g. care setting, model of delivery, intensity and timing of intervention) | N | N | N | N | N | N | P | N | N | N | 0 |
| 8 | Boundaries of the analysis (defining the scope or comprehensiveness of the study) | N | N | N | N | N | N | N | N | N | N | 0 |
| 9 | Time horizon | Y | Y | Y | Y | Y | Y | N | Y | Y | Y | 9 |
| 10 | Analytic perspectives (e.g., Reference Case perspectives included [healthcare sector, societal]; other perspectives such as employer or payer) | Y | Y | Y | Y | Y | Y | N | Y | Y | P | 8 |
| 11 | Whether this analysis meets the requirements of the Reference Case | N | N | N | N | N | N | N | N | N | N | 0 |
| 12 | Analysis plan | P | P | P | P | P | Y | P | Y | Y | Y | 4 |
|  |  |  |  |  |  |  |  |  |  |  |  |  |
|  | **Methods and data** |  |  |  |  |  |  |  |  |  |  |  |
| 13 | Trial- based analysis or model- based analysis. | Y | Y | Y | Y | Y | Y | Y | Y | Y | Y | 10 |
| 14 | Description of event pathway/ model (describe condition or disease and the health states included) | Y | Y | P | Y | Y | Y | Y | P | P | Y | 7 |
| 15 | Diagram of event pathway/ model (depicting the sequencing and possible transitions among the health states included) | Y | Y | Y | Y | Y | Y | Y | Y | Y | Y | 10 |
| 16 | Description of model used (e.g., decision tree, state transition, microsimulation) | Y | Y | Y | Y | Y | Y | Y | Y | Y | Y | 10 |
| 17 | Modelling assumptions | Y | Y | P | Y | Y | P | P | Y | Y | Y | 7 |
| 18 | Software used | Y | Y | Y | Y | Y | Y | Y | Y | Y | Y | 10 |
| 19 | Identification of key outcomes | Y | Y | Y | Y | Y | Y | Y | Y | Y | Y | 10 |
| 20 | Complete information on sources of effectiveness data, cost data, and preference weights | Y | Y | N | Y | Y | Y | P | P | Y | Y | 7 |
| 21 | Methods for obtaining estimates of effectiveness including approach(es) used for evidence synthesis | N | N | P | P | N | N | N | Y | N | Y | 2 |
| 22 | Methods for obtaining estimates of costs and preference weights | P | N | N | N | N | N | P | P | P | Y | 1 |
| 23 | Critique of data quality | P | N | N | N | N | Y | N | N | Y | Y | 3 |
| 24 | Statement of costing year; this is the year to which all costs have been adjusted for the analysis (e.g., 2016) | Y | Y | Y | N | Y | Y | Y | Y | Y | Y | 9 |
| 25 | Statement of method used to adjust costs for inflation | Y | N | N | N | N | Y | N | N | N | Y | 3 |
| 26 | Statement of type of currency | Y | Y | Y | Y | Y | Y | Y | Y | Y | Y | 10 |
| 27 | Source and methods for obtaining expert judgment, if applicable | N | P | NA | NA | NA | N | N | NA | NA | NA | 0 |
| 28 | Statement of discount rate(s) | Y | Y | Y | Y | NA | NA | N | N | Y | Y | 6 |
|  |  |  |  |  |  |  |  |  |  |  |  |  |
|  | **Results** |  |  |  |  |  |  |  |  |  |  |  |
| 29 | Results of model validation | N | N | N | N | N | N | N | N | N | N | 0 |
| 30 | Reference Case results (discounted and undiscounted): total costs and effectiveness, incremental costs and effectiveness, incremental cost-effectiveness, ratios, measure(s) of uncertainty | P | N | N | N | P | N | N | N | N | N | 0 |
| 31 | Disaggregated results for important categories of costs, outcomes, or both | N | N | Y | Y | Y | Y | N | N | N | N | 4 |
| 32 | Results of sensitivity analysis | P | Y | P | P | P | Y | P | P | P | P | 2 |
| 33 | Other estimates of uncertainty | N | N | N | N | N | N | N | N | N | N | 0 |
| 34 | Graphical representation of cost- effectiveness results | N | Y | N | N | N | N | N | N | N | N | 1 |
| 35 | Graphical representation of uncertainty analyses | Y | Y | N | P | Y | Y | N | Y | N | Y | 6 |
| 36 | Aggregate cost and effectiveness information | N | N | N | N | N | N | N | N | N | N | 0 |
| 37 | Secondary analyses | Y | Y | N | N | N | N | N | N | N | Y | 3 |
|  |  |  |  |  |  |  |  |  |  |  |  |  |
|  | **Discussion** |  |  |  |  |  |  |  |  |  |  |  |
| 38 | Summary of Reference Case results | P | N | N | N | N | N | N | N | N | N | 0 |
| 39 | Summary of sensitivity of results to assumptions and uncertainties in the analysis | Y | Y | Y | Y | N | Y | Y | Y | Y | Y | 9 |
| 40 | Discussion of the study results in the context of results of related CEAs | Y | Y | Y | Y | N | Y | Y | Y | Y | Y | 9 |
| 41 | Discussion of ethical implications (e.g., distributive implications relating to age, disability, or other characteristics of the population) | N | N | N | N | N | N | N | N | N | N | 0 |
| 42 | Limitations of the study | Y | Y | P | N | Y | Y | N | Y | Y | Y | 7 |
| 43 | Relevance of study results to specific policy questions or decisions | Y | Y | Y | Y | Y | Y | Y | Y | Y | Y | 10 |
| 44 | **Conclusions** | Y | Y | Y | Y | Y | Y | Y | Y | Y | Y | 10 |
|  |  |  |  |  |  |  |  |  |  |  |  |  |
| **45** | **Technical appendix** | N | N | N | N | N | N | N | N | N | Y | 1 |
|  |  |  |  |  |  |  |  |  |  |  |  |  |
|  | **Disclosures** |  |  |  |  |  |  |  |  |  |  |  |
| 46 | Statement of any potential conflicts of interest relating to funding source, collaborations, or outside interests | N | Y | N | N | N | Y | Y | Y | N | N | 5 |
|  | **Impact Inventory** |  |  |  |  |  |  |  |  |  |  |  |
| 47 | Full accounting of consequences within and outside of the healthcare sector (refer below) | N | N | P | P | N | N | N | P | N | N | 0 |
|  | Total items rated “Yes” | 26 | 28 | 19 | 22 | 21 | 27 | 16 | 24 | 24 | 31 | - |
|  | % items rated “Yes” | 55.3 | 59.6 | 40.4 | 46.8 | 44.7 | 57.4 | 34.0 | 51.1 | 51.1 | 66.0 | - |
|  | Total items rated “Partial” | 6 | 2 | 8 | 5 | 4 | 3 | 7 | 5 | 3 | 2 |  |
|  | % items rated “Partial” | 12.8 | 4.3 | 17.0 | 10.6 | 8.5 | 6.4 | 14.9 | 10.6 | 6.4 | 4.3 |  |
|  |  |  |  |  |  |  |  |  |  |  |  |  |
|  | Total items rated “No” | 15 | 17 | 19 | 19 | 20 | 16 | 24 | 17 | 19 | 13 |  |
|  | % items rated “No” | 31.9 | 36.2 | 40.4 | 40.4 | 42.6 | 34.0 | 51.1 | 36.2 | 40.4 | 27.7 |  |
|  |  |  |  |  |  |  |  |  |  |  |  |  |
|  | Total items rated “Not Applicable” | 0 | 0 | 1 | 1 | 2 | 1 | 0 | 1 | 1 | 1 |  |
|  | % items rated “Not Applicable” | 0.0 | 0.0 | 2.1 | 2.1 | 4.3 | 2.1 | 0.0 | 2.1 | 2.1 | 2.1 |  |
|  |  |  |  |  |  |  |  |  |  |  |  |  |

*Each item is rated “Yes”, “Partial”, “No” or “Not Applicable”.*

# Appendix 9 Methodological quality ratings of included studies, based on the CHEC-extended checklist

*Reminder: to update the reference numbers*

|  | **Items** | **[10]** | **[11]** | **[12]** | **[13]** | **[14]** | **[15]** | **[16]** | **[17]** | **[18]** | **[19]** | **Total Y / RY** |
| --- | --- | --- | --- | --- | --- | --- | --- | --- | --- | --- | --- | --- |
| 1 | Is the study population clearly described? |  |  |  |  |  |  |  |  |  |  | 8 |
| 2 | Are competing alternatives clearly described? |  |  |  |  |  |  |  |  |  |  | 3 |
| 3 | Is a well-defined research question posed in answerable form? |  |  |  |  |  |  |  |  |  |  | 10 |
| 4 | Is the economic study design appropriate to the stated objective? |  |  |  |  |  |  |  |  |  |  | 10 |
| 5 | Are the structural assumptions and the validation methods of the model properly reported? |  |  |  |  |  |  |  |  |  |  | 1 |
| 6 | Is the chosen time horizon appropriate in order to include relevant costs and consequences? |  |  |  |  |  |  |  |  |  |  | 7 |
| 7 | Is the actual perspective chosen appropriate? |  |  |  |  |  |  |  |  |  |  | 6 |
| 8 | Are all important and relevant costs for each alternative identified? |  |  |  |  |  |  |  |  |  |  | 8 |
| 9 | Are all costs measured appropriately in physical units? |  |  |  |  |  |  |  |  |  |  | 10 |
| 10 | Are costs valued appropriately? |  |  |  |  |  |  |  |  |  |  | 9 |
| 11 | Are all important and relevant outcomes for each alternative identified? |  |  |  |  |  |  |  |  |  |  | 8 |
| 12 | Are all outcomes measured appropriately? |  |  |  |  |  |  |  |  |  |  | 10 |
| 13 | Are outcomes valued appropriately? |  |  |  |  |  |  |  |  |  |  | 6 |
| 14 | Is an appropriate incremental analysis of costs and outcomes of alternatives performed? |  |  |  |  |  |  |  |  |  |  | 10 |
| 15 | Are all future costs and outcomes discounted appropriately? |  |  |  |  |  |  |  |  |  |  | 6 |
| 16 | Are all important variables, whose values are uncertain, appropriately subjected to sensitivity analysis? |  |  |  |  |  |  |  |  |  |  | 8 |
| 17 | Do the conclusions follow from the data reported? |  |  |  |  |  |  |  |  |  |  | 10 |
| 18 | Does the study discuss the generalizability of the results to other settings and patient/client groups? |  |  |  |  |  |  |  |  |  |  | 2 |
| 19 | Does the article indicate that there is no potential conflict of interest of study researcher(s) and funder(s)? |  |  |  |  |  |  |  |  |  |  | 4 |
| 20 | Are ethical and distributional issues discussed appropriately? |  |  |  |  |  |  |  |  |  |  | 0 |
|  | Total items rated “Y / RY” | 14 | 17 | 13 | 12 | 11 | 13 | 12 | 15 | 13 | 16 | - |
|  | % items rated “Y / RY” | 70.0 | 85.0 | 65.0 | 60.0 | 55.0 | 65.0 | 60.0 | 75.0 | 65.0 | 80.0 | - |
|  |  |  |  |  |  |  |  |  |  |  |  |  |
|  | Total items rated “N / RN” | 5 | 3 | 7 | 8 | 8 | 7 | 6 | 4 | 7 | 4 | - |
|  | % items rated “N / RN” | 25.0 | 15.0 | 35.0 | 40.0 | 40.0 | 35.0 | 30.0 | 20.0 | 35.0 | 20.0 | - |
|  |  |  |  |  |  |  |  |  |  |  |  |  |
|  | Total items rated “Unclear” | 1 | 0 | 0 | 0 | 1 | 0 | 2 | 1 | 0 | 0 | - |
|  | % items rated “Unclear” | 5.0 | 0.0 | 0.0 | 0.0 | 5.0 | 0.0 | 10.0 | 5.0 | 0.0 | 0.0 | - |
|  |  |  |  |  |  |  |  |  |  |  |  |  |

| *Each item is shaded* |  | *for “Yes / Rather Yes (Y / RY)* |  | *for “No / Rather No (N / RN)” or* |  | *for “Unclear (UC)”.* |
| --- | --- | --- | --- | --- | --- | --- |

**References:**

1. Hoffmann, T.C., P.P. Glasziou, I. Boutron, R. Milne, R. Perera, D. Moher, et al., *Better reporting of interventions: template for intervention description and replication (TIDieR) checklist and guide.* BMJ : British Medical Journal, 2014. **348**: p. g1687.

2. Neumann, P.J., T.G. Ganiats, L.B. Russell, G.D. Sanders and J.E. Siegel, *Cost-Effectiveness in Health and Medicine*. 2 ed. 2016, New York: Oxford University Press. 536.

3. Sanders, G.D., P.J. Neumann, A. Basu, D.W. Brock, D. Feeny, M. Krahn, et al., *Recommendations for Conduct, Methodological Practices, and Reporting of Cost-effectiveness Analyses: Second Panel on Cost-Effectiveness in Health and Medicine.* Jama, 2016. **316**(10): p. 1093-103.

4. Evers, S.M., M.E.J.B. Goossens, d.H.C.W. Vet, v.M. Tulder and A. Ament, *Criteria list for assessment of methodological quality of economic evaluations:consensus on health economic criteria.* International Journal of Technology Assessessment in Health Care, 2005.

5. Odnoletkova, I., G. Goderis, L. Pil, F. Nobels, B. Aertgeerts, L. Annemans, et al., *Cost-Effectiveness of Therapeutic Education to Prevent the Development and Progression of Type 2 Diabetes: Systematic Review.* Journal of Diabetes and Metabolism, 2014. **5**(9).

6. Watts, R.D. and I.W. Li, *Use of Checklists in Reviews of Health Economic Evaluations, 2010 to 2018.* Value in Health, 2019. **22**(3): p. 377-382.

7. Nixon, J., K.S. Khan and J. Kleijnen, *Summarising economic evaluations in systematic reviews: a new approach.* BMJ (Clinical research ed.), 2001. **322**(7302): p. 1596-1598.

8. Higashi, M.K. and D.L. Veenstra, *Managed care in the genomics era: assessing the cost effectiveness of genetic tests.* The American journal of managed care, 2003. **9**(7): p. 493-500.

9. Zhu, Y., K.M. Swanson, R.L. Rojas, Z. Wang, J.L. St. Sauver, S.L. Visscher, et al., *Systematic review of the evidence on the cost-effectiveness of pharmacogenomics-guided treatment for cardiovascular diseases.* Genetics in Medicine, 2020. **22**(3): p. 475-486.

10. Eckman, M.H., S.K. Singh, J.K. Erban and G. Kao, *Testing for Factor V Leiden in Patients with Pulmonary or Venous Thromboembolism: A Cost-Effectiveness Analysis.* Medical Decision Making, 2002. **22**(2): p. 108-124.

11. Rubio-Terrés, C., J.M. Soria, P.E. Morange, J.C. Souto, P. Suchon, J. Mateo, et al., *Economic Analysis of Thrombo inCode, a Clinical–Genetic Function for Assessing the Risk of Venous Thromboembolism.* Applied Health Economics and Health Policy, 2015. **13**(2): p. 233-242.

12. Marchetti, M., S. Quaglini and G. Barosi, *Cost‐effectiveness of screening and extended anticoagulation for carriers of both factor V Leiden and prothrombin G20210A.* QJM: An International Journal of Medicine, 2001. **94**(7): p. 365-372.

13. Marchetti, M., A. Pistorio and G. Barosi, *Extended anticoagulation for prevention of recurrent venous thromboembolism in carriers of factor V Leiden--cost-effectiveness analysis.* Thromb Haemost, 2000. **84**(5): p. 752-7.

14. You, J.H.S., F.W.H. Chan, R.S.M. Wong and G. Cheng, *The potential clinical and economic outcomes of pharmacogenetics-oriented management of warfarin therapy – a decision analysis.* Thromb Haemost, 2004. **92**(09): p. 590-597.

15. You, J., K. Tsui, R. Wong and G. Cheng, *Potential Clinical and Economic Outcomes of CYP2C9 and VKORC1 Genotype-Guided Dosing in Patients Starting Warfarin Therapy.* Clinical Pharmacology & Therapeutics, 2009. **86**(5): p. 540-547.

16. Schalekamp, T., G.J.J. Boink, L.E. Visser, B.H.C. Stricker, A. de Boer and O.H. Klungel, *CYP2C9 genotyping in acenocoumarol treatment: Is it a cost-effective addition to international normalized ratio monitoring?* Clinical Pharmacology & Therapeutics, 2006. **79**(6): p. 511-520.

17. O'Brien, S.H. and K.J. Smith, *Using Thrombophilia Testing to Determine Anticoagulation Duration in Pediatric Thrombosis is not Cost-Effective.* The Journal of Pediatrics, 2009. **155**(1): p. 100-104.

18. Auerbach, A.D., G.D. Sanders and J. Hambleton, *Cost-effectiveness of testing for hypercoagulability and effects on treatment strategies in patients with deep vein thrombosis.* The American Journal of Medicine, 2004. **116**(12): p. 816-828.

19. Simpson, E.L., M.D. Stevenson, A. Rawdin and D. Papaioannou, *Thrombophilia testing in people with venous thromboembolism: systematic review and cost-effectiveness analysis.* Health Technol Assess, 2009. **13**(2): p. iii, ix-x, 1-91.
